# A novel gene cluster allows preferential utilization of fucosylated milk oligosaccharides in *Bifidobacterium longum* subsp. *longum* SC596

**DOI:** 10.1038/srep35045

**Published:** 2016-10-19

**Authors:** Daniel Garrido, Santiago Ruiz-Moyano, Nina Kirmiz, Jasmine C. Davis, Sarah M. Totten, Danielle G. Lemay, Juan A. Ugalde, J. Bruce German, Carlito B. Lebrilla, David A. Mills

**Affiliations:** 1 Department of Viticulture & Enology, University of California, One Shields Ave. Davis, CA 95616, United States; 2 Department of Foods for Health Institute and University of California, One Shields Ave. Davis, CA 95616, United States; 3 Department of Chemical and Bioprocess Engineering, School of Engineering, Pontificia Universidad Catolica de Chile, Av. Vicuña Mackenna 4860, Santiago, Chile; 4 Department of Chemistry, University of California, One Shields Ave. Davis, CA 95616, United States; 5 Genome Center, University of California, One Shields Ave. Davis, CA 95616, United States; 6 Centro de Genetica y Genomica, Facultad de Medicina, Clinica Alemana, Universidad del Desarrollo, Santiago, Chile; 7 Department of Food Science & Technology, University of California, One Shields Ave. Davis, CA 95616, United States

## Abstract

The infant intestinal microbiota is often colonized by two subspecies of *Bifidobacterium longum*: subsp. *infantis* (*B. infantis*) and subsp. *longum* (*B. longum*). Competitive growth of *B. infantis* in the neonate intestine has been linked to the utilization of human milk oligosaccharides (HMO). However, little is known how *B. longum* consumes HMO. In this study, infant-borne *B. longum* strains exhibited varying HMO growth phenotypes. While all strains efficiently utilized lacto-*N-*tetraose, certain strains additionally metabolized fucosylated HMO. *B. longum* SC596 grew vigorously on HMO, and glycoprofiling revealed a preference for consumption of fucosylated HMO. Transcriptomes of SC596 during early-stage growth on HMO were more similar to growth on fucosyllactose, transiting later to a pattern similar to growth on neutral HMO. *B. longum* SC596 contains a novel gene cluster devoted to the utilization of fucosylated HMO, including genes for import of fucosylated molecules, fucose metabolism and two α-fucosidases. This cluster showed a modular induction during early growth on HMO and fucosyllactose. This work clarifies the genomic and physiological variation of infant-borne *B. longum* to HMO consumption, which resembles *B. infantis*. The capability to preferentially consume fucosylated HMO suggests a competitive advantage for these unique *B. longum* strains in the breast-fed infant gut.

The human gut microbiome is a complex metabolic ‘organ’ that significantly impacts human health^1^. The assembly of this ‘organ’ starts immediately after birth, when various microbial clades from different body sites are introduced into the neonate and begin a long-term relationship with the host^2^. It is generally understood that variables such as diet, mode of birth and early-life stresses have a profound influence on the successions of gut microbes during the neonatal period^3^,^4^. These patterns of colonization, in turn, have been shown by certain studies to impact health later in life^5^. In general, the infant gut microbiome exhibits a characteristic composition during nursing often dominated by *Bifidobacterium* species^6^ prior to transition into a more adult-like composition of species, influenced by the introduction of solid foods^7^.

Of special interest is how breastfeeding can shape the gut microbiome of infants. In addition to providing critical nutrients and bioactive molecules that support growth and development^8^,^9^, breast milk contains complex oligosaccharides that escape intestinal enzymes and transit through the gastrointestinal tract of the infant^10^. Free human milk oligosaccharides (HMO) are present in high concentrations in milk^8^, suggesting that their function is important even though they have little nutritional value for the infant. These carbohydrates are far from simple: they range from 3 to 32 in their degree of polymerization, and are composed of five main monosaccharides: D-glucose (Glc), D-galactose (Gal), *N-*acetylglucosamine (GlcNAc), L-fucose (Fuc) and *N-*acetylneuraminic (or sialic) acid (Neu5Ac). Complexity in HMO lies in the diversity of glycosidic bonds in these molecules, rendering a large number of potential combinations^11^,^12^. While the total concentration of HMO appears to be tightly regulated, variation in their abundance, is explained in part by host genetic factors^8^.

In spite of this complexity, a relatively small number of HMO species can represent up to 70% of the total^12^,^13^, including fucosylated neutral HMO such as 2′-fucosyllactose (2FL), 3-fucosyllactose (3FL) and lacto-*N-*fucopentaoses^12^; acidic HMO such as sialyllacto-*N-*tetraoses, 3′- and 6′-sialyllactose (3SL and 6SL)^12^, and neutral non-fucosylated HMO such as lacto-*N-*tetraose (LNT), lacto-*N*-neotetraose (LNnT) and lacto-*N-*hexaose (LNH)^14^.

Comparisons of intestinal microbiota of breast-fed and formula-fed infants reveal a strong correlation between breastfeeding and the overabundance of *Bifidobacterium* spp.^15^. These bacteria can represent up to 90% of the total gut bacteria during breastfeeding^16^, and their abundance decreases later in life^15^. Bifidobacteria are generally recognized as beneficial, with several species currently used in probiotic formulations. Certain bifidobacteria are more common in infants, such as *Bifidobacterium longum* subsp. *infantis* (*B. infantis*) and *B. breve*. Other species seem to be present both in the infant and adult gut microbiota, such as *B. bifidum*, *B. pseudocatenulatum* and *Bifidobacterium longum* subsp. *longum* (*B. longum*)^17^,^18^.

*B. infantis* and *B. bifidum* have been examined as two different models for milk glycan consumption^19^. *B. infantis* internalizes HMO, relying on several ABC transporters with defined specificity for certain HMO families^20^, followed by the action of numerous glycosyl hydrolases to metabolize milk glycans^21^,^22^,^23^,^24^. Recently, select *B. breve* isolates that grow well on HMO were shown to internalize and metabolize complex fucosylated HMO^25^. In contrast*, B. bifidum* has an array of cell-wall associated glycosyl hydrolases^26^,^27^, enabling HMO degradation outside the cell and select mono- and disaccharides imported via specific transporters for catabolism^28^. Notably, the mode of consumption by *B. bifidum* leaves fucose and sialic acid externally, monomers that can subsequently serve as growth substrates for other bacteria^29^,^30^,^31^. Indeed, *B. bifidum* was recently shown to release mucin-associated sialic acid which cross fed *B. breve* UCC2003^32^.

*B. longum* strains are also present at high concentrations in the infant gut microbiota^15^,^17^. However, several genomic and functional studies have indicated this subspecies is more adapted to an adult diet^33^,^34^, possessing transporters and glycosyl hydrolases active on plant oligosaccharides. Despite membership within the same species, genomes of *B. longum* are notably different from *B. infantis*^35^, with the latter possessing a wealth of genes associated to consumption of milk glycans, suggesting a lifestyle more adapted to the milk-fed infant gut^36^.

HMO consumption has only been examined in a few *B. longum* strains to date, where the consumption profile appears rather restricted by comparison to the *B. infantis* subspecies^37^,^38^. While genomic^35^ and phenotypic^39^ screens of multiple *B. infantis* strains indicate that strong growth on HMO is a signature of the subspecies, HMO consumption is more variable in strains from other bifidobacterial species^25^. Given the importance of the early assembly of the infant gut microbiota on long term health, it is important to understand which microbes are uniquely enriched by milk glycans and examine the genetic functions responsible for this enrichment. In this study we demonstrate unique genetic loci are responsible for vigorous growth on fucosylated HMO by select isolates of *B. longum* and present genomic and functional characterization of HMO consumption by a prototypical strain, *B. longum* SC596.

## Results

### Genetic characterization of *B. longum* isolates by MLST

In a previous screen of bifidobacterial isolates obtained from breast-fed infants^25^, 297 *B. longum* species group isolates were further identified as *B. longum* subsp. *infantis* or subsp. *longum* using Multilocus Sequencing Typing (MLST) analysis^40^. A total of 158 isolates were identified as *B. infantis* and 139 as *B. longum* by MLST (data not shown). The MLST analysis of this pool of *B. longum* strains resolved into a library of 17 *B. longum* individual strain types (Table S1). A MLST-based phylogenetic tree of these strains combined with additional *B. longum* isolates obtained from various culture collections and other *B. longum* strains for which a genome sequence was available is shown in Figure S1. Interestingly, four of these strains are from adult origin (Table S1), however they were not differentiable from infant strains (Figure S1), suggesting infant and adult strains of *B. longum* are genomically similar. In addition, MLST data suggests that *B. longum* subsp. *infantis* 157F, a strain that has been shown to inhibit enteropathogenic *Escherichia coli* in an animal model^41^, is actually a *B. longum* subsp. *longum* strain. This is consistent with the 157F genome lacking several gene clusters characteristic of *B. infantis* subspecies^33^,^34^.

### Physiology of the consumption of HMO in *B. longum*

To understand the strain-level growth behavior of *B. longum* on HMO, we used HMO purified from pooled breast milk samples, in addition to individual HMO structures, as the sole carbon source in growth experiments. The individual HMO structures represent the chemical diversity found in breast milk: LNT, LNnT, 2FL, 3FL, 3SL and 6SL. We also included mucin as a substrate, as it has been reported that some *B. longum* strains can use these glycoproteins as a carbon source^42^.

Most *B. longum* strains grew to intermediate levels on HMO (OD_600_ values (0.5–0.8); Table 1 and Figure S2), values which are lower than that obtained by *B. infantis*^37^. Growth using specific HMO structures showed important differences in HMO utilization among the strains. All strains grew well on LNT (Galβ1-3GlcNAc linkage; Figure S2) as the sole carbon source (Table 1). LNT is the most abundant oligosaccharide in breast milk that supports the growth *in vitro* of several bifidobacteria^38^. In contrast, growth of *B. longum* strains on the isomer LNnT (containing a Gal(β1-4)GlcNAc linkage) was markedly low for most strains, with the exception of SC558 and SC664 that displayed good growth on this oligosaccharide (Table 1, Figure S2).

**Figure 1:**
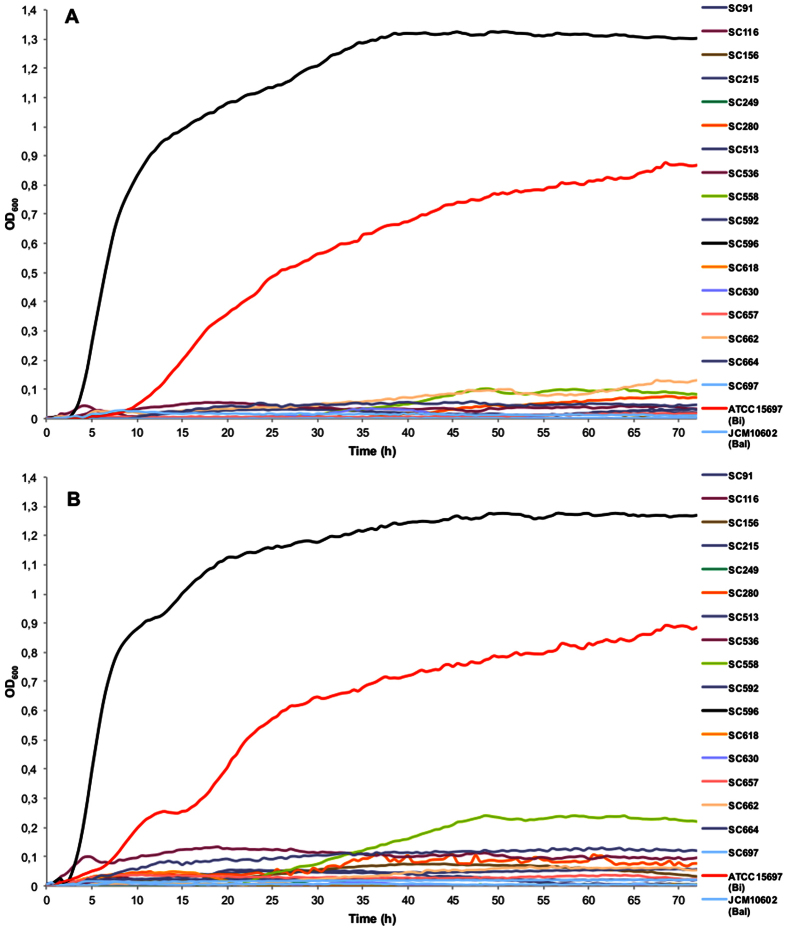
Growth of *B. longum* isolates on mMRS medium supplemented with 2% 2FL (**A**) or 3FL (**B**). Growth was measured as OD of the media at 600 nm. Fermentations were carried out in triplicate; controls consisted of inoculated medium lacking of substrates and un-inoculated medium containing a substrate which was also used as blank for OD measurements. Bi: *B. infantis*, Bal: *B. animalis*.

Fucosylated HMO represent a large percentage in breast milk, and utilization of these carbohydrates is common in *B. bifidum*, *B. infantis*, and select *B. breve* strains^22^,^25^,^38^. Using 2FL and 3FL as a carbon source, we found that most *B. longum* strains do not grow on these substrates (Table 1). However, *B. longum* strain SC596 exhibited vigorous growth on 2FL and 3FL, reaching maximum OD_600_ values considerably higher than *B. infantis* (Fig. 1A,B).

None of the *B. longum* strains were able to grow on 3SL or 6SL as a sole carbon source (Table 1, Figure S2). In contrast, several strains grew at lower levels on mucin as the sole carbon source, higher than *B. animalis* but not similar to known mucin-degrader *B. bifidum* PRL2010^28^ (Table 1 and Figure S2). Strain SC215 displayed higher OD than any other *B. longum* isolate on this substrate (Figure S2). Mucin appears to be preferentially used by several *B. bifidum* strains^28^, and several *B. longum* and *B. bifidum* strains have been shown to encode endo-α-*N-*acetylgalactosaminidases^42^,^43^, enzymes that can release *O*-linked glycans from intestinal mucins.

Maximum growth OD_600_ values for each *B. longum* strain on each substrate were examined via principal component analysis (PCA; Figure S3). PCA1 and PCA2 explain 53.59% and 21.12% of data variation, respectively. This analysis indicates that in general the patterns of utilization of LNT, LNnT, and mucin were the most diverse among *B. longum*. The second part of the plot shows that strain SC596 is rather divergent from the rest of strains, which is probably due to its better growth on several classes of HMO. In contrast, strain SC249 showed poor growth on all substrates tested and also appeared distant from the other strains. In summary, the ability of *B. longum* to grow on individual HMO appears strain-specific, with several novel strains consuming complex HMO.

### Glycoprofiling of the HMO consumption

**Figure 2:**
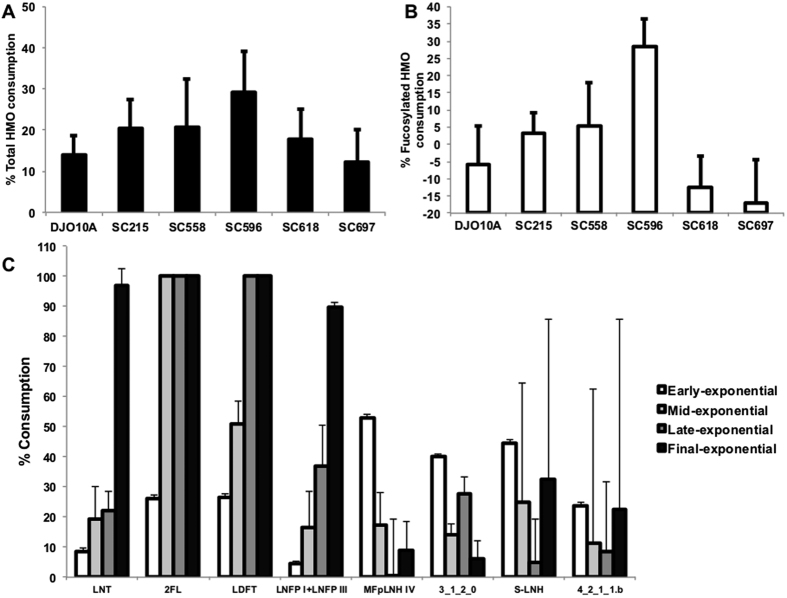
Glycoprofiling of the HMO consumption by select *B. longum* strains. (**A**) Total utilization of HMO; (**B**) Total fucosylated HMO consumption. Bacteria were incubated with pooled HMO from breast milk and consumption was calculated as the percent difference in HMO between the start and the end of exponential phase. (**C**) Temporal glycoprofile of the consumption of select neutral and acidic HMO by *B. longum* SC596 at different phases during the exponential phase. Error bars represent experiments in triplicate.

To better understand HMO consumption preferences, mass spectrometry-based glycoprofiling was run on strains that displayed better growth on HMO (SC215, SC596, SC558, SC618 and SC697; Table 1), with *B. longum* DJO10A as a control. Accounting for total HMO consumption, percentages were rather low, varying from 15 to 30% for strain SC596 (Fig. 2A). *B. longum* DJO10A and JCM1217 have been the only strains of this subspecies previously tested for HMO growth, and their low consumption is in agreement with these results (Fig. 2A). Utilization of fucosylated HMO was significantly higher for strain SC596 (30%), with consumption also observed for strains SC215 and SC558 (Fig. 2B).

**Figure 3:**
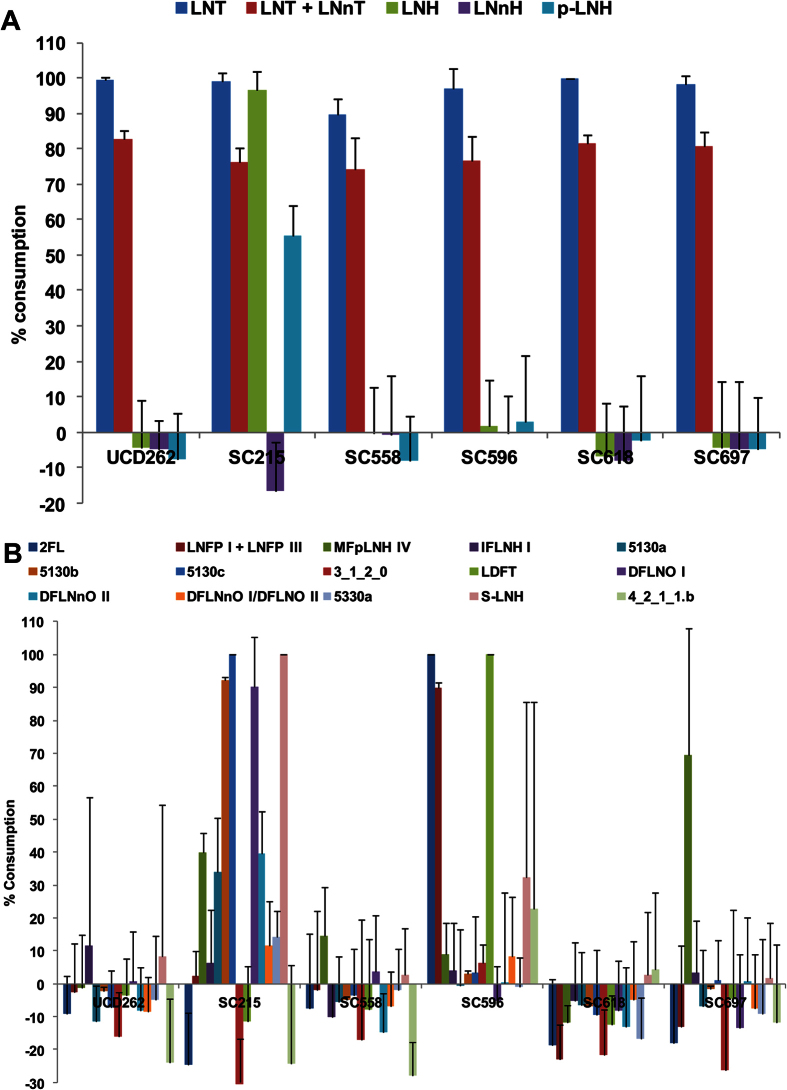
Glycoprofiling HMO consumption on select *B. longumw* strains. (**A**) Percentage of utilization of neutral HMO; (**B**) Percentage of utilization of fucosylated and sialylated HMO. Bacteria were incubated with pooled HMO from breast milk and consumption was calculated as the percent difference in HMO between the start and the end of exponential phase. Error bars represent experiments in triplicate.

Details on specific HMO structure consumption are shown in Fig. 3, and detailed HMO compositions are shown in Table S2. All strains tested completely consumed LNT (and/or its isomer, LNnT, which cannot be resolved from the analysis), in agreement with the idea that LNT is a preferred substrate for *B. longum*. Strain SC215 was unique in that it could ferment LNH, its isomer p-LNH, and sialyl-LNH (Fig. 3A) as well as several larger, complex fucosylated HMO (Fig. 3B). The ability of SC215 to degrade complex fucosylated HMO is interesting considering this strain cannot use 2FL as a growth substrate (Figure S2). In agreement with the FL growth phenotype, *B. longum* SC596 completely depleted 2FL, LNFP and LDFT from the HMO pool (Fig. 3B).

Temporal glycoprofiling of HMO by SC596 revealed fucosylated HMO are utilized first, with 2FL and LDFT reaching 100% consumption during mid exponential growth (Fig. 2C). In contrast, neutral HMO like LNT were depleted only in late exponential phase. Utilization of LNFP I and III followed a similar pattern as LNT and consumption of other HMO did not show any temporal preference. In aggregate, these results indicate that specific infant *B. longum* strains consume fucosylated HMO, and that one strain actually has a preference for fucosylated HMO over neutral oligosaccharides.

### Genome sequencing of *B. longum* SC596

Due to the increased consumption of fucosylated HMO by *B. longum* SC596, a draft genome sequence was generated in order to identify key genes linked to this enhanced HMO consumption. To sequence SC596, 45 million reads with HiSeq Illumina sequencing (100 bp paired ends, coverage 800X) were generated, and the Integrated Microbial Genome Expert Review annotation platform ( https://img.jgi.doe.gov, GOLD Project ID: Gi17164), was used for annotation and deposition. The SC596 genome assembled into 50 contigs, with an average of 52092 bp per contig. Genome size was 2.602 Mb, with a G + C content of 59.93%.

### Comparative genomics of subsp. *longum*

The genome of *B. longum* SC596 contains a variety of genes associated with oligosaccharide utilization (Table S3). In particular, *B. longum* SC596 was found to contain two genes encoding α-fucosidases, similar to *B. longum* strains CMCC P0001 and JDM301 (Table S3). These three same strains also possessed an overabundance of the COG1653 (Family 1 Solute Binding Proteins) compared to other *B. longum* genomes (Table S3). These proteins are the binding component of ABC transporters for oligosaccharides. These observations initially suggest that SC596 has an additional preference for complex oligosaccharides by comparison to the other *B. longum* strains.

Phylogenomic level comparison of *B. longum* genomes suggest strain SC596 is more related to strains from adult origin, than other infant strains (Figure S4). Comparative genomics also confirm that *B. longum* 157F belongs to the subspecies *longum* and that *B. longum* JDM301 forms a different cluster—observations which are in agreement with the MLST data (Figure S1) and previous *B. longum* pangenome analysis^34^. *B. longum* DJO10A, a strain from adult origin and one of the most studied in the species *longum*, does not grow well on HMO^37^. A direct comparison of DJO10A and SC596 (Figure S5) revealed genes unique to each strain suggesting differential adaptations. For example, strain SC596 contains a complete cluster for exopolysaccharide biosynthesis, which is absent in DJO10A. It also contains a two-component system and a potassium channel with a putative role in osmoregulation (data not shown). Strain SC596 also possesses several other ABC transporters for oligosaccharides compared to DJO10A. Genes from DJO10A plasmids, pDOJH10-L and pDOJH10-S, were absent in strain SC596, as well as a gene encoding a NADPH-quinone reductase.

### Global transcriptomics of *B. longum* SC596 during growth on HMO

The transcriptomic response of SC596 on HMO was explored in two sets of experiments. SC596 was grown on HMO purified from pooled breast milk samples as the sole carbon source, and samples were taken at four time points during exponential phase (early, mid1, mid2 and late exponential, see materials and methods). These samples match those used for glycoprofiling described above. In addition, *B. longum* SC596 was grown to mid exponential phase on select individual HMO: LNT, LNnT, 2FL and 3FL. Samples taken during growth on lactose were used as a reference control, given that this sugar is the structural precursor of all HMO, and that *B. longum* uses lactose instead of glucose as the preferential carbon source^44^.

Over 163 million 50 bp reads were generated from 36 independent RNA-seq experiments with an average of 9.09 million reads per sample using Illumina HiSeq (Table S4). Of these, 76.6% aligned to coding genes, with an 11.6% aligning to intergenic regions. 16S and 23S transcripts represented in average 0.9% and 2.3% of the total sequences. Other features and statistics of these experiments are shown in Table S4.

**Figure 4:**
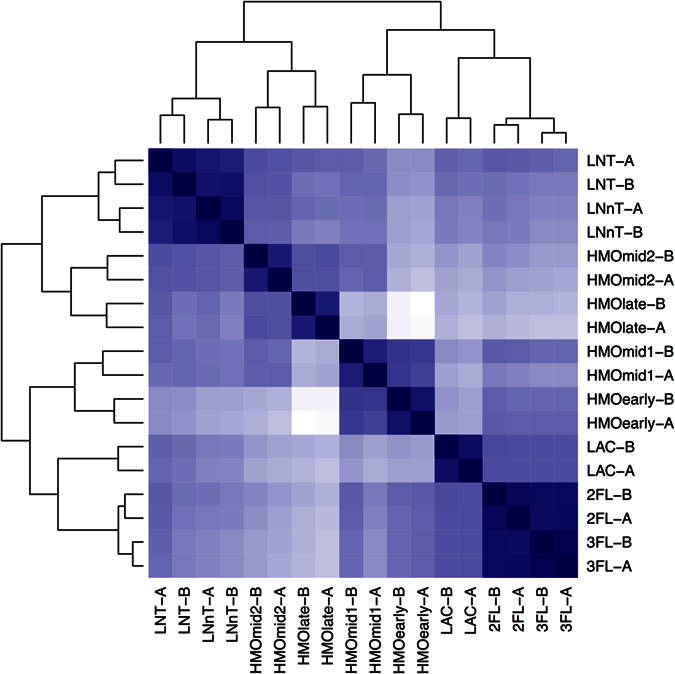
Global transcriptome distances in substrate responses of *B. longum* SC596 to human milk oligosaccharides. The heatmap shows the distances between the whole transcriptomes in response to LNT, LNnT, lactose (LAC), 2FL, 3FL, and four time points during HMO growth (early, mid1, mid2, late). Each experiment on each substrate was done in duplicate (i.e. samples ‘A’ and ‘B’). Branches represent the Euclidean distances of the whole transcriptomes after variance-stabilization of the count data. The intensity of the blue color in the heatmap indicates the degree of similarity, from white (dissimilar) to dark blue (most similar). Likewise, the dendograms also show the distances with larger branch lengths indicating greater distances.

All datasets were first compared among each other. The heatmap in Fig. 4 shows the Euclidian distances between the whole transcriptome of each sample. This analysis shows that all replicates are most similar to each other. Interestingly, global responses of *B. longum* SC596 to 2FL and 3FL were very similar and more related to lactose than any HMO time point. Transcriptomes from the early and mid1 time points during growth on HMO clustered closer to lactose and FLs, while mid2 and late HMO time points appeared more similar to LNT and LNnT. Out of all the substrates, the HMO early and late time points were the most different (Fig. 4), suggesting a large change in transcriptional state in strain SC596 during growth on HMO, complementing the progression in oligosaccharide preferences depicted in Fig. 2C.

#### Differentially Expressed Genes

A large number of genes were differentially regulated by each treatment relative to lactose (Tables S5, S6 and S7). These genes were classified by their log2foldchange relative to lactose, and analyzed using Gene Set Enrichment Analysis. Functional annotations tested included COG functions, KEGG pathways, GO terms, and SRI pathways.

Functional enrichment results for genes up- and down-regulated relative to lactose are summarized in Tables S5 and S6, respectively. Several gene categories related to translational processes were significantly up-regulated at the early HMO points. Categories such as ATPases of ABC transporters and carbohydrate transport were upregulated during growth on certain HMO species. Interestingly, growth on HMO also induced xylan utilization genes (Table S6). Other evidence has indicated a role for this pathway in utilization of host-derived glycans by *B. bifidum*^28^.

#### Functional transcriptomics of HMO utilization in B. longum SC596

Integration of genomic information and transcriptomic profiles during growth on pooled HMO or single HMO molecules allowed identification of several clusters likely responsible for the unusual HMO consumption phenotype of SC596. To complement these observations, we examined the biological function of the ABC transporters and enzymes in these clusters, by using a mammalian glycan array to determine the affinities of the transporters or mass spectrometry to determine the specificities of glycosyl hydrolases for HMO.

#### B. longum LNB/GNB cluster

**Figure 5:**
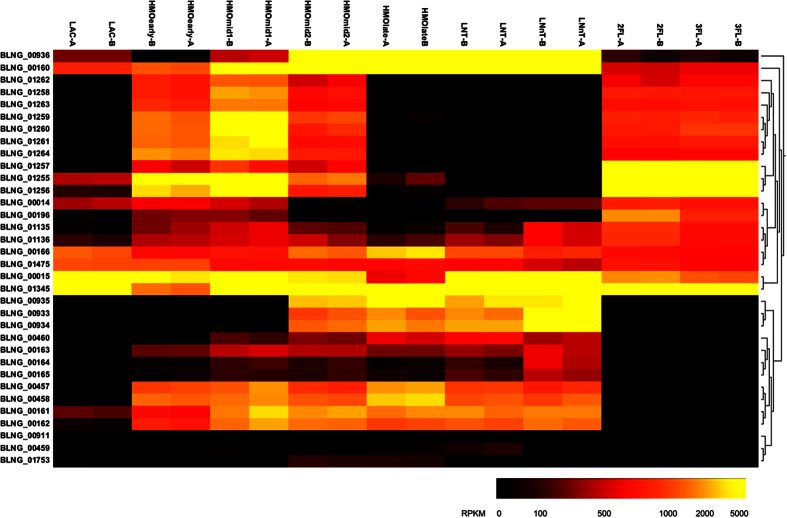
Hierarchical clustering of features of carbohydrate metabolism genes induced in*B*. longum SC596 **during growth on HMO used in this study (See** **Table S7**). Heat map shows intensity of these genes in response to LNT, LNnT, lactose (LAC), 2FL, 3FL, and four time points during HMO growth (early, mid1, mid2, late). Each experiment on each substrate was done in duplicate (i.e. samples ‘A’ and ‘B’). Clustering is calculated according to RPKM values for each gene.

Most bifidobacterial genomes studied to date contain a cluster devoted to the import and processing of type 1 HMO (termed the LNB/GNB cluster^46^, Figure S6). This cluster contains an ABC importer for LNT and other HMO containing the Galβ1-3GlcNAc/GalNAc linkage^20^, and a Leloir-like pathway that further process galactose and GlcNAc/GalNAc into the bifid shunt^47^. In SC596 all genes in this cluster (BLNG_00160-00166; Table S8) were induced several fold during growth on HMO, suggesting their participation in HMO metabolism (Fig. 5). These genes were also induced several fold during growth on LNT and LNnT as well. However, levels of expression were low during growth on lactose, 2FL or 3FL, which is in agreement with the idea of their specific role in metabolism of HMO containing the Galβ1-3GlcNAc linkage.

Solute Binding Proteins (SBPs) are a major determinant of specificity in the import of complex oligosaccharides in bifidobacteria^20^. The SBP BLNG_00160 in the LNB/GNB cluster binds linkages with Galβ1-3 (Galβ1-3Gal/GlcNAc/GalNAc; Table 2), as found in LNT, Galacto-*N-*tetraose (GNT), LNB and GNB. In addition, BLNG_00160 showed high affinity for other structurally related glycans, such as sialyl LNT, LNT, and even several complex bi- and tri-antennary *N-*glycans (Table 2). Several milk and intestinal glycoproteins contain *N-*glycans, which have been thought to be potential carbon sources for intestinal bifidobacteria^48^.

A second ABC transporter (BLNG_00933-00936; Figure S6) was also induced up to 300 fold during growth on HMO, however only at later time points (Fig. 5). This cluster was also highly expressed during growth on LNT and LNnT again, suggesting a linkage to the LNT consumption preferences witnessed in Fig. 2C. The glycan specificity of the SBP in this cluster (BLNG_00936) is oriented toward oligosaccharides with Galβ1-3 as common motif (Galβ1-3GalNAc, GNB and Galβ1-3Galβ1-4GlcNAc), albeit via weak binding (Table 2).

#### B. longum β-galactosidases

In general, β-galactosidases active on HMO have a preference for either type 1 (Gal(β1-3)GlcNAc), or type 2 (Gal(β1-4)GlcNAc) linkages found in HMO^21^. In *B. longum* SC596 only two genes encoding β-galactosidases (BLNG_00015, BLNG_01753) were expressed at considerable values at all conditions assessed in this study (Fig. 5, Table S8). BLNG_00015 belongs to the carbohydrate active enzyme (CAZY) family GH2 while BLNG_01753 is a member of the GH42 family (Table S8). Neither enzyme appeared to be extracellular since they lack transmembrane domains or signal peptide sequences. Their amino acid sequences share more than 95% identity with Blon_2334 and Blon_2016 (respectively) from *B. infantis* ATCC 15697; previously studied enzymes that display high efficiency towards lactose and LNnT (type 2; Blon_2334), and LNT-like HMO (type 1; Blon_2016)^21^,^49^.

**Figure 6:**
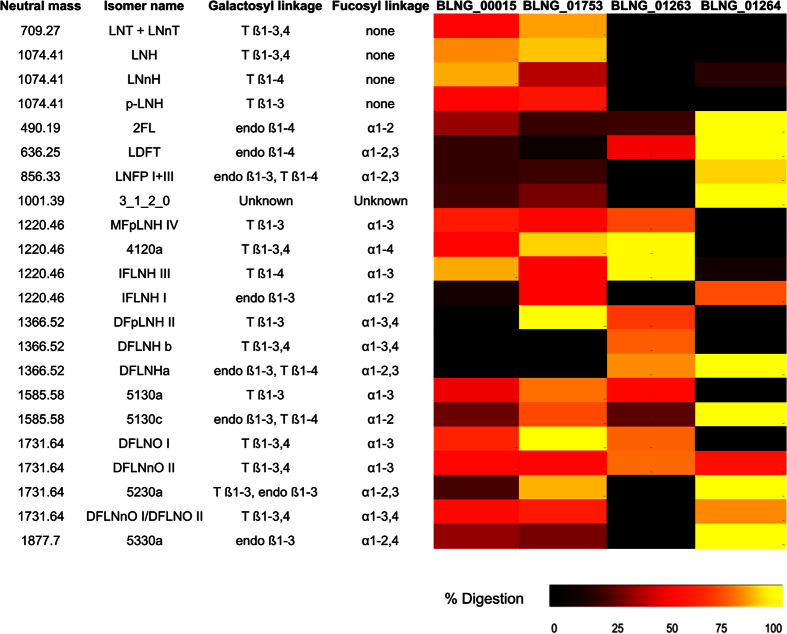
Heat map of the digestion of individually-quantified oligosaccharides from pooled HMO, by β-galactosidases (BLNG00015 and BLNG01753) and α-fucosidases (BLNG01263 and BLNG01264) purified from *B. longum* SC596). Enzymes were incubated with pooled HMO and products were quantified using LC-MS. The percentage digestion calculated for each of the specific oligosaccharide degraded by these enzymes is expressed via a heat map. HMO names and structures were reported in ref. 12. T: Terminal linkage; endo: internal linkage.

Considering that all substrates used in transcriptome profiling contain a terminal lactose, it is not unexpected that BLNG_00015 displayed high expression values on all substrates used and at any time point during growth on HMO (with a decrease at the late-exponential phase; Fig. 5). Purified BLNG00015 enzyme exhibited maximal activity at pH 6.0 and 55 °C. This enzyme appeared to be specific for type 2 linkages releasing galactose from lactose and LNnT (TLC results, data not shown). Mass spectrometry was also used to detect the activity of this enzyme previously incubated with HMO purified from breast milk (Fig. 6). BLNG00015 displayed a general preference for terminal β1-4 linkages, as found in LNH, LNnH, and other more complex oligosaccharides up to 10 residues, although the enzyme reduced the concentration of several other HMO (Fig. 6).

A second β-galactosidase involved in HMO degradation in *B. longum* SC596 is BLNG_01753, a GH42 family enzyme. Expression of BLNG_01753 displayed medium RPKM values (150–500 RPKM) across all substrates, but with increasing values toward later time points during growth on HMO. This observation correlates with neutral HMO being fermented after fucosylated HMO observed during the temporal glycoprofiling (Fig. 5). Purified BLNG01753 has an optimum pH of 5.5 and 55 °C, similar to what has been found in its homolog in *B. infantis*^49^. Mass spectrometry-based analysis of HMO cleavage products revealed BLNG01753 displayed a general preference for type 1 HMO species as found in LNT, LNH, DFpLNH II and DFLNO I (Fig. 6). Together, these data indicate that neutral HMO are metabolized by *B. longum* SC596, with features that resemble more of the *B. infantis* strategy for HMO consumption (import of HMO and intracellular degradation mediated by SBPs) than that of *B. bifidum* (extracellular degradation and import of mono and disaccharides^19^).

#### B. longum FHMO cluster

**Figure 7:**
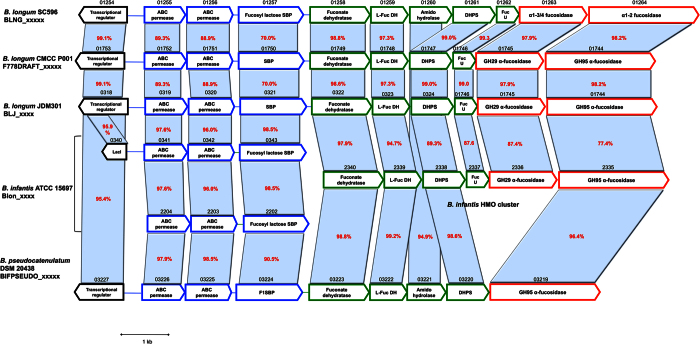
FHMO utilization cluster in *B. longum* SC596, and homologous genes in bifidobacteria. Arrows represent genes, and numbers on top of each gene indicate the locus tag number in the respective genome. Text inside the arrows indicates predicted or validated annotations. Red numbers indicate percent identity between corresponding genes and homologs relative to strain SC596. Colors of each gene are indicative of the primary function of each respective gene: transcriptional regulators (black), oligosaccharide transport (blue), carbohydrate feeder pathways (green) and glycosyl hydrolases (red). SBP: Solute Binding Protein; L-Fuc DH: L-fuconate dehydrogenase; DHPS: Dihydropicolinate synthase; FucU: L-fucose mutarotase.

Strain consumption data described above revealed that SC596 has a preference for fucosylated HMO such as 2FL and LNFP. Genome analysis and RNA-seq revealed a prominent gene cassette in SC596 that was absent in DJO10A, a strain that does not consume fucosylated oligosaccharides (Fig. 7 ref. 37). This cluster, termed FHMO (Fucosylated Human Milk Oligosaccharides utilization cluster) comprises genes BLNG_01254 to BLNG_01264, encoding a LacI-like transcriptional regulator, an ABC transporter, an SBP and ABC permease system, putative fucose metabolism genes and two α-fucosidases (Fig. 7).

The putative SBP in the FHMO cluster, BLNG_01257, provides the glycan specificity to the ABC cassette. Purified BLNG01257 bound strongly to Fuc(α1-2)Gal, the H-antigen precursor in ABO glycans, and also a common epitope in milk oligosaccharides (Table 2). This SBP was also able to recognize 2FL and Fuc (α1-2) Gal (β1-4) GlcNAc(β1-3) GalNAc. BLNG_1257 is homolog to Blon_0343 and Blon_2202 in *B. infantis*, SBPs that display identical ligand affinities for small fucosylated HMO^20^.

The two α-fucosidases present in the FHMO cluster (BLNG_01263 and BLNG_01264, Fig. 7) appear to be intracellular enzymes, given the lack of transmembrane domains or signal peptide sequences. Recombinant BLNG01263 displayed a preference for α(1–3/4) Fuc linkages, digesting HMO up to 10 residues such as IFLNH III, MFpLNH IV and DFLNO I and II (Fig. 6). These results resemble α-fucosidase Blon_2336, a close homolog found in *B. infantis* ATCC 15697^22^. The second α-fucosidase on this cluster, BLNG_01264, shares a 77% identity with Blon_2335 in *B. infantis*^22^. Purified BLNG01264 was shown to digest α(1–2) fucosyl linkages, however it also cleaved certain HMO with a α(1–3) Fuc linkage (Fig. 6). For example, it removed nearly 100% 2′FL, LDFT, LNFP and DFLNHa, all containing an α(1–2) fucosyl linkages. Interestingly, the specificities of BLNG_01263 and BLNG_01264 are highly complementary, indicating that expression of both enzymes possibly renders 100% consumption for most fucosylated HMO (Fig. 6).

All the genes within the FHMO cluster were expressed in a coordinated fashion across all conditions suggesting a single transcriptional unit (Fig. 5). Expression of the FHMO cluster was induced nearly 100 fold relative to lactose during growth on 2′FL or 3FL (Fig. 5) however the cluster was not expressed during growth on lactose, LNT or LNnT, substrates not containing fucose. Induction of the cluster peaked during growth at earlier time points during growth on HMO, returning to basal levels in late exponential phase. This observation correlates with the preferential consumption of fucosylated HMO by *B. longum* SC596 by comparison to neutral HMO (Fig. 2C).

An examination of other sequenced *B. longum* strains identified similar FHMO clusters in two *B. longum* strains, one of them isolated from a healthy infant (Fig. 7; ref. 50). Interestingly, the FHMO cluster found has significant homology to two ABC transporters in *B. infantis* (Blon_0341-0343 and Blon_2202-2204), and part of the well-described HMO cluster I in *B. infantis* (Fig. 7; ref. 33).

## Discussion

Bifidobacteria are important members of the intestinal microbiome in both infants and adults^4^. HMO and other milk glycoconjugates are considered the primary substrates for enrichment of these species in the infant gut. Previous genomic evidence suggests that *B. infantis* co-evolved with mammalian milk glycans via a complement of genes that allow the strains to be competitive in consumption of HMO in the neonate gastrointestinal tract^33^. These genetic determinants, forming discrete gene clusters in *B. infantis*, are mostly absent in *B. longum* strains of adult origin^35^. Indeed, *B. longum* isolated from adults has been usually regarded to be more competitive on plant oligosaccharides^51^. In this study, we determined that select *B. longum* strains can robustly consume HMO and identified and characterized gene clusters associated with this activity in strain SC596.

The HMO utilization in *B. longum* has only been addressed in a few adult-derived strains: DJO10A, JCM1217 and ATCC 15707^37^,^38^,^52^. These strains exhibited poor HMO metabolism, only consuming LNT from the pool HMO species present in human milk. In this study, we isolated and characterized a larger panel of infant-derived *B. longum* strains, and demonstrated more variation in growth on HMO. In addition to LNT utilization, which is mostly conserved in infant and adult-borne bifidobacteria, we found a *B. longum* strain displaying vigorous growth on LNnT (SC664, Figure S2). In addition to *B. longum* SC596 (that was further characterized in this study), strain SC215 displayed also a very competitive phenotype in HMO utilization in addition to mucin growth. This strain was unique in that it consumed neutral hexaoses such as LNH and p-LNH (Fig. 3), and several fucosylated HMO such as MFpLNH IV, 5130 a, 5130 b and DFLNO I (Fig. 3 ref. 12). The inability of SC215 to grow or consume 2FL, compared to more complex fucosylated HMO, suggests that 2FL is imported by different transporters. Moreover, SC215 also consumed vigorously sialyl-LNH (Fig. 3). While we did not find any evidence for sialidase activity on this strain, it is possible that this oligosaccharide is still imported and the neutral part of the molecule might be utilized. In aggregate, these observations allow us to conclude that HMO utilization is also variable in *B. longum*, similar to what has been observed for *B. breve* and *B. bifidum*^25^,^53^, and in contrast with *B. infantis* strains that display a very consistent consumption of major types of HMO^37^.

*B. longum* SC596 was able to grow vigorously on 2FL and 3FL to higher OD_600_ values than reference strain *B. infantis* ATCC 15697. When growing on pooled HMO, SC596 exhibited a clear preference for fucosylated HMO such as 2FL and LNFP, while consuming neutral HMO later during *in vitro* growth. Temporal glycoprofiling has shown different results among bifidobacteria. While *B. infantis* does not exhibit a preference for any neutral or fucosylated HMO consuming several HMO simultaneously^22^, specific *B. breve* strains are able to deplete sialylated HMO before reaching full consumption of neutral HMO such as LNT^25^. The genetic control mechanisms responsible for these different HMO species preferences are yet unknown, however the presence of LacI-like transcriptional regulators in several of the clusters described suggests they control the hierarchy in HMO utilization.

Whole transcriptomes of *B. longum* SC596 during exponential growth on HMO were largely divergent, indicating a large transcriptional change in SC596 as different HMO species were catabolized (Fig. 4). Such transition was not previously noted in *B. infantis* HMO-transcriptomes^53^, which were rather stable during growth, or *B. bifidum* SC555, where HMO-transcriptomes were similar to neutral HMO (LNT and LNnT). The latter strain focused its metabolism in this class of HMO rather than sialylated or fucosylated HMO^53^.

SC596 transcriptomes during growth on 2FL and 3FL were more similar to those observed at the early stages of growth on HMO, which is in agreement with the observed preference for fucosylated HMO determine by glycoprofiling and the induction of the FHMO cluster. Similarly, SC596 transcriptome transition in late exponential growth on HMO to a pattern similar to growth on LNT and LNnT, is again indicative of the prioritization of fucosylated oligosaccharides by SC596 over neutral HMO species.

Genomic and transcriptomic analyses helped identify the genetic loci putatively responsible for growth of *B. longum* SC596 on HMO. HMO utilization strategies in SC596 appear to occur via a mechanism that seems very similar to *B. infantis* ATCC 15697, which is based on the activity of several ABC transporters for HMO and intracellular glycosyl hydrolases. For example, the SBP in the LNB/GNB cluster (BLNG_00160) from SC596 displayed similar affinities for type 1 HMO as its homologs in *B. infantis* and *B. longum* strains^20^,^54^. Growth on HMO and other single HMO species led to a high expression of β-galactosidases and α-fucosidases that are genetically and functionally similar to previously characterized enzymes in *B. infantis* ATCC 15697^22^,^49^. One important consequence of this mode of consumption is that does not allow cross-feeding with other gut species, since oligosaccharides are imported inside the cell with each monosaccharide being fermented intracellularly.

Although full expression of the LNB/GNB cluster occurred during late exponential phase growth on HMO in addition to LNT and LNnT, it is yet unclear how LNT is metabolized. The activation of the LNB/GNB cluster, in particular genes encoding lacto-*N-*biose phosphorylase, *N-*acetylhexosamine 1-kinase and GalE and GalT (Fig. 5 and Table S8), indicates that LNB is actively metabolized into the Leloir pathway^47^. How LNB is formed and is taken inside the cell is not yet clear. One possibility is that a membrane-bound lacto-*N-*biosidase cleaves LNT into LNB and lactose. However, SC596 does not appear to possess any homolog for this enzyme. It has been recently found that *B. longum* JCM1217 encodes a novel membrane lacto-*N-*biosidase^55^, which is enzymatically different from the enzyme previously characterized in *B. bifidum*^56^. This enzyme was not found in other *B. longum* genomes examined in this study excepting strain BBMN68.

Alternatively, degradation of neutral HMO could be sequential, using exo-acting glycosyl hydrolases. The activities of β-galactosidases and β-hexosaminidases release Gal, Glc and GlcNAc. While a β-hexosaminidase in SC596 displayed medium-low RPKM values across substrates (BLNG_00911; RPKM~100), expression data suggests that GlcNAc is metabolized in *B. longum* SC596 by two major enzymes: *N-*acetylglucosamine-6-phosphate deacetylase and glucosamine-6-phosphate isomerase (BLNG_00457 and BLNG_00458). The respective genes are located in a cluster together with a transcriptional regulator (BLNG_00459) and a sugar kinase (BLNG_00460; Figure S6); a homolog of a similar gene in *B. infantis* we previously hypothesized activates GlcNAc to GlcNAc-6-P^24^. BLNG_00457 and BLNG_00458 increase their expression 45–50 times during growth on HMO, LNT or LNnT relative to lactose, 2FL or 3FL. The putative sugar kinase (BLNG_00460) also displays a 10-fold increase in its expression on these substrates (Fig. 5).

One striking difference between *B. longum* strains that do not grow well on HMO (such as DJO10A) and SC596 was the presence of the FHMO cluster. Affinities of the cognate SBP, and specificities of the two encoded α-fucosidases from this cluster clearly indicated their involvement in consumption of fucosylated HMO. In addition, the FHMO cluster was expressed as an HMO-inducible unit, in a modular fashion on fucosylated substrates and not during growth on neutral HMO. Fucose is a key monosaccharide characteristic of host-derived glycans, and it plays roles in pathogenesis and serves as a carbon source for certain gut microbes^57^. For example, *Bacteroides thetaiotaomicron* α-fucosidases release fucose in the gut environment rendering it available for other bacteria^58^. How fucose is metabolized in bifidobacteria is yet unclear however, the high expression of putative fucose metabolizing enzymes (Fig. 5; Table S8) suggest that fucose is used as a carbon source in bifidobacteria endowed with this pathway.

The FHMO cluster in *B. longum* SC596 displays some similarities with the HMO cluster I from *B. infantis* (BLNG_01258 to BLNG_01264; ref. 33), with nearly identical fucose metabolism genes and α-fucosidases (Fig. 7). Moreover, the FHMO cluster displays homology with two ABC cassettes (SBPs and permeases) found in other regions of the ATCC 15697 genome, that putatively import 2FL and Fuc(α1-2)Gal^20^. This suggests that these clusters are genetically related, however establishing ancestry is difficult given the relatedness of the strains. Considering that the intact HMO cluster I appears to be conserved within *B. infantis*^33^,^35^, it is tempting to hypothesize that portions of the HMO cluster I transferred to select *B. longum* isolates residing in the infant gut microbiota.

## Conclusion

In this work we examined a panel of *B. longum* isolates of infant origin to determine the physiological and genetic variation for HMO consumption. Unlike *B. infantis*, the growth phenotype of infant-borne *B. longum* strains on HMO appears variable and only select strains are capable in consuming large, complex HMO species. Further characterization of a *B. longum* strain that grows well on fucosylated HMO revealed a unique preference for these structures over neutral HMO. Genomic analysis identified key gene clusters linked to fucosylated HMO consumption, resembling cognate genes in *B. infantis* ATCC 15697, however *B. longum* SC596 displayed a unique and modular induction of HMO utilization genes. The characterization of *B. infantis*^19^, *B. breve*^25^ and *B. longum* isolates with unique HMO consumption preferences suggests a complementary strategy for survival in the nursing infant gut whereby different species and subspecies may be able to gain an advantage by more effectively consuming a targeted portion of the HMO repertoire delivered to the infant colon.

## Methods

### Bacteria and media

*Bifidobacterium* strains used in this study (Table S1) were previously obtained from fecal samples from exclusively breast-fed term infants and deposited in the UC Davis Viticulture & Enology Culture Collection (Davis, CA^25^). Bacteria were also obtained from the Japanese Collection of Microorganisms (Riken Biosource Center Japan), the American Type Culture Collection (Manassas, VA). For routine experiments, bifidobacteria were grown on de Man-Rogose-Sharp (MRS) broth supplemented with 0.05% w/v L-cysteine (Sigma-Aldrich, St. Louis, MO), and incubated for 18 h at 37 °C in an anaerobic chamber (Coy Laboratory Products, Grass Lake, MI), in an atmosphere containing 5% carbon dioxide, 5% hydrogen, and 90% nitrogen. Prior to each assay all bacteria were subcultured twice.

### Multilocus sequence typing (MLST) of strains

MLST analysis of *B. longum* strains targeted intragenic regions of seven housekeeping genes *clpC, purF, gyrB, fusA, Iles, rplB, rpoB* were selected based on a previous study^25^. PCR amplification and analysis was carried as in ref. 25. Cycling conditions were optimized for every primer set (Table S9). Sequencing was performed on an ABI 3730 Capillary Electrophoresis Genetic Analyzer using BigDye Terminator chemistries at the University of California Davis DNA Sequencing Facility. Sequencing data for all loci was edited using BioEdit 7.0 and aligned using CLUSTAL W^59^. Phylogenetic analysis and concatenations of the sequenced loci were performed using the Molecular Evolutionary Genetic Analysis (MEGA) software version 5 ( http://megasoftware.net). Descriptive evolutionary analysis including mol % G + C content, number of polymorphic sites, nucleotide diversity π/site, average number of nucleotide differences k were calculated using DnaSP version 5.10. Allelic sequences were assigned as described previously^55^. A minimum evolution tree of the concatenated loci was calculated using MEGA 5.0 (Figure S1).

### Bifidobacterial growth *in vitro* on HMO

The 17 *B. longum* strains in Table S1 were tested for growth in the presence of seven different substrates as the sole carbon source: HMO from pooled breast milk^60^, LNT, lacto-*N-*neotetraose (LNnT), 2′-fucosyllactose (2FL), 3-fucosyllactose (3FL) (generously provided by Glycom, Denmark), 6′-sialyllactose (6′SL) (generously provided by GenChem Inc. Korean), and hog mucin type II (Sigma). *B. animalis* subsp*. lactis* JCM 10602 was also included as negative control and *B. infantis* ATCC 15697 and *B. bifidum* PRL2010 as positive controls for growth experiments. Two μl of each resulting overnight culture were used to inoculate 200 μl of modified MRS medium (mMRS), devoid of glucose and supplemented with 2% (w/v) of each substrate, except for mucin at 1%, as the sole carbohydrate source, and another 2 μl inoculated into mMRS without added sugar. The media was supplemented with 0.05% (w/v) L-cysteine, and in all the cases the cultures in the wells of the microtiter plates were covered with 30 μl of sterile mineral oil to avoid evaporation. The incubations were carried out at 37 °C in an anaerobic chamber (Coy Laboratory Products, Grass Lake, MI). Cell growth was monitored in real time by assessing optical density (OD) at 600 nm using a BioTek PowerWave 340 plate reader (BioTek, Winoosky, VT) every 30 min preceded by 15 seconds shaking at variable speed. Two biological replicates and three technical replicates were performed for every studied strain. The OD obtained for each strain grown on the different substrates was compared with the OD obtained in the absence of a sugar source. This difference in OD (ΔOD) was used as a parameter to evaluate each strain’s ability for growing on the different substrates.

### Statistical analysis of growth

Statistical analysis of the data was carried out using SPSS for Windows, 15.0 (SPSS Inc Chicago, IL, USA). The relationships among the maximum OD_600_ values of the growth on the different substrates by the bifidobacteria strains were evaluated by Pearson correlation coefficients and principal components analysis (PCA).

### Glycoprofiling

Bacterial cultures in mMRS with 2% HMO were collected at the end of the exponential phase and centrifuged at 12000 × *g* for 30 min. In the case of *B. longum* SC596, the samples were collected at four different points in the growth curve, approximately OD_600nm_ = 0.2, 0.4, 0.6 and 0.75. At least two biological replicates were performed in triplicates. Glycoprofiling was performed as in ref. 25. Supernatants were filtered using a multiscreen 96-well filtration plate 0.22 μm (Millipore, Billerica, MA) prior to storage at −80 °C. Remaining oligosaccharides were recovered from the supernatants (25 μl) and reduced to their alditol forms with 1M NaBH_4_ at 65 °C for 1.5 h. Each replicate was desalted by solid-phase extraction on graphitized carbon cartridges. Salts were removed with 6 mL of deionized water and oligosaccharides were eluted with 20% acetonitrile in water (v/v) and with 40% acetonitrile in 0.01% trifluoroacetic acid (v/v). Solid-phase extraction fractions were combined and dried under vacuum. Samples were reconstituted in 100 μL of deionized water and diluted 50-fold for LC-MS analysis.

An Agilent nano-high performance liquid chromatography-chip/-time-of-flight (nano-HPLC-Chip/TOF) mass spectrometer equipped with a capillary pump for sample loading and a nano pump for chromatographic separation was used for HMO analysis. Separation was performed on a microfluidic chip equipped with an enrichment and nano-LC analytical column, both packed with porous graphitized carbon, using a previously described method^12^,^61^. Briefly, HMO were separated by a 60 min gradient using a binary solvent system consisting of 3% acetonitrile/water in 0.1% formic acid (v/v) solvent A and 90% acetonitrile/water in 0.1% formic acid (v/v) solvent B. HMO were analyzed in positive ion mode, with a mass range between *m/z* 300–2000. Agilent’s MassHunter software was used for data acquisition and data analysis version B.03.01.

HMO monosaccharide composition was determined using accurate mass within ±20 ppm mass error of theoretically calculated masses. Specific structures were assigned to HMO peaks by matching the reproducible retention time to that reported in previously annotated HMO libraries^11^,^61^. Percent consumption was calculated using a label-free method, employing the uninoculated HMO pool as an external standard. Total HMO consumption was calculated with respect to the uninoculated control by normalizing the summed abundance of all identified oligosaccharide spectra in ion counts in the bacterial supernatant to that of the control using equation (1):


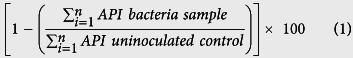


where API is absolute peak intensity and n is the number of identified HMO. The consumption of individual HMO species was quantitated in the same manner, in which the absolute peak intensity of a specific HMO structure was normalized to the peak intensity of the corresponding structure in the un-inoculated control.

### Genome sequencing

*B. longum* SC596 was grown on MRS broth under the anaerobic conditions described above. Genome sequencing was performed as in ref. 53. Briefly, DNA was extracted using the MasterPure Gram-positive DNA Purification Kit (Epicentre), and sonicated in a Bioruptor Standard (Diagenode, Denville NJ). Fragmented cDNA was used to prepare a library for Illumina sequencing using the Apollo 324 Robot and the PrepX ILM DNA Library Kit (IntegenX, Pleasanton CA), at the Genome Center at UC Davis. Sample was ligated with BiooScientific Adapter 13 (Bioo Scientific, Austin TX). Finally, sample was pooled with other seven bacterial genomes at the Genome Center at UC Davis DNA Technologies Core Facility, for sequencing on a Illumina HiSeq2500. All sequencing steps were carried out by Core personnel. Sequencing was run for 200 cycles, with read length of 100 bp (paired ends). Sequencing files were edited for quality using CLC-Bio Genomics Workbench (Cambridge, MA), and aligned to the genome sequence of *B. longum* DJO10A^62^. Resulting contigs from the genome of *B. longum* SC596 was annotated via IMG Expert Review (IMG/ER) (GOLD card: Gi17164. ER submission ID: 8631). Locus tags generated contained the BLNG prefix, and topology was linear. The gene calling method used was the Isolate Genome Gene Calling. Draft genome sequences were compared using the IMG/ER functions, and IMG/ER was also used on genomic analyses. The final draft genome of *B. longum* SC596 is available at https://img.jgi.doe.gov, GOLD Project ID: Gi17164.

### Clustering analysis

All the genomes were downloaded from the IMG database^63^. To obtain the set of shared and unique proteins between the genomes, an all-vs-all BlastP^64^ was performed, and the results were processed using the OrthoMCL pipeline, using an inflation value of 1.5. The results were parsed using python scripts ( https://github.com/juanu/CompMicroGenom), which allowed us to identify the set of shared and unique proteins for each genome.

### Phylogenomic analysis

A total of 953 single-copy gene clusters were processed to generate a phylogenomic tree of all the organisms. First, all clusters were aligned using Mafft^65^, and then checked for evidence of recombination using Phipack^66^. A total of 275 clusters were selected, and all the alignments were concatenated. This large alignment matrix was trimmed using Gblocks^67^, with a resulting alignment length of 202,423 nt. The phylogenetic tree was generated using FastTree (Price, Dehal, & Arkin, 2010) with the GTR model.

### RNA-seq

*B. longum* SC596 was grown as described above in mMRS supplemented with either 2% lactose, 2% HMO, 2% LNT, 2% LNnT, 2% 2FL, or 2% 3FL in a microplate reader, and cultures were taken at mid-exponential phase OD 0.5–0.6. In the case of HMO, the samples were collected at four different points in the growth curve, approximately OD_600 nm_ = 0.2, 0.4, 0.6, and 0.75. Samples were immediately pelleted at 12000 × *g* for 1 min and stored in RNA later (Ambion). RNA extraction, ribosomal RNA depletion and cDNA conversion were performed as previously described^53^. Briefly, RNA was obtained using the Ambion RNAqeous kit (LifeTechnologies), with a pretreatment using 250 μl lysozyme (50 mg/ml final; Sigma) and 125 μl mutanolysin (1000 units/ml; Sigma), at 37 °C for 10 min. Total RNA was immediately subjected to DNase treatment with the Turbo DNase free (Ambion) using 3 μl of DNase I for 1 hour at 37 °C. RNA integrity was checked in a Bioanalyzer using an Agilent RNA Nano Chip, with a minimal RIN (RNA Integrity Number) of 7. Genomic DNA contamination was evaluated by qPCR and universal bacterial primers Uni334F and Uni514R^53^. Ribosomal RNA depletion was achieved using the Ribo-Zero magnetic kit Bacteria (Epicentre, Madison WA). Manufacturer instructions were followed (using 28 or 26 μl of RNA depending on the concentration). mRNA was purified with the Qiagen RNeasy Minelute Cleanup kit, following the instructions presented in the Ribo-Zero protocol for this part. RNA was eluted in 13 μl. One μl of the RNA was checked for removal of 16S and 23S rRNA peaks using the Qubit HS RNA Assay (Agilent). Chromatograms were manually inspected. Messenger RNA was finally converted to cDNA. For first strand cDNA synthesis, the Superscript II Reverse Transcriptase was used (Invitrogen) and second cDNA strand was synthesized using the NEBNext mRNA Second Strand Synthesis Module (New England Biolabs, Ipswich, MA). Then, the MinElute Reaction Cleanup Kit (Qiagen, Valencia CA) was used for cleaning up the DNA. cDNA was quantified using the Qubit High Sensitivity DNA kit (Life Technologies), and fragmented in a Bioruptor (Diagenode). An average fragment length of 300-500 bp was desired for next steps.

Sequencing libraries were prepared using the BiooScientific NEXTflex Chip Seq Kit (Bioo Scientific, Austin TX), similar to ref. 53. This kit is Illumina compatible and with RNA-seq applications. After Bioanalyzer and Qubit checks, libraries were molarly pooled and submitted to the Genome Center at UC Davis Genome Center DNA Technologies Core Facility, for sequencing on an Illumina HiSeq2500. All sequencing steps were carried out by Core personnel. Sequencing was run for 50 cycles, with read length of 50 bp (single reads). Runs were demultiplexed by the UC Davis Genome Center DNA Technologies Core Facility.

Reads were processed as in ref. 53 using the CLC-Bio Genomics Workbench. Reads were trimmed setting at 2 the maximum of ambiguous base pairs, and deleted if their quality scores were below 0.5. Edited reads were aligned to fasta files generated at IMG from the genome sequence (reference) of *B. longum* subsp. *longum* SC596. Only coding regions were considered in this analysis. Alignment allowed a maximum of 2 mismatches, and a maximum of 10 matches to other reads. Other settings were considered as default. The CLC-BIO tools retrieved information regarding the gene length, total and unique gene reads aligning to a specific locus tag, and automatically calculated the RPKM value (Reads Per Kilobase per Million mapped reads). Statistical analysis such as hierarchical clustering were performed using RPKM data for each sample as in ref. 53, as well as Differential Expression determination using the R package “DESeq” and Gene Set Enrichment Analysis. The data from the *B. longum* SC596 RNA-sequencing studies are available in the NCBI Gene Expression Omnibus database (http://www.ncbi.nlm.nih.gov/geo/) with accession number GSE87697.

### Gene cloning and protein expression

Gene sequences were analyzed using the Integrated Microbial Genomes (IMG) database. For glycosyl hydrolase coding genes, primers were designed with modifications for cloning using the pEco-T7-cHis cloning kit (Gentarget Inc) and for SBP coding genes, primers were designed with modifications for cloning using the pEco-T7-nGST cloning kit (Gentarget Inc) Primer sequences are shown in Table S9. Genomic DNA from *B. longum* SC596 was purified from a 1 ml culture using the Qiagen DNeasy Blood & Tissue Kit following the manufacturer’s instructions for purification of Gram Positive bacterial DNA. BLNG_01753, BLNG_01264, and BLNG_01263 genes were amplified by PCR using 0.8 uM of each forward and reverse primer (Table S9), 42 ng genomic DNA, 0.2 mM GeneAmp deoxynucleotide triphosphate mix (Applied Biosystems, Foster City, CA), and 0.5 U of Phusion High-Fidelity DNA Polymerase (New England Biolabs, Ipswich, MA) in a 25 μl final volume. BLNG_00015 was amplified by PCR using 0.75 uM of each forward and reverse primer, 86 ng genomic DNA, 0.18 mM GeneAmp deoxynucleotide triphosphate mix (Applied Biosystems, Foster City, CA), and 1 U of Phusion High-Fidelity DNA Polymerase (New England Biolabs, Ipswich, MA) in a 53 μl final volume. For glycosyl hydrolases, BLNG_01753, BLNG_01264, and BLNG_01263, PCR was conducted with initial denaturation of 98 °C for 30 s and 35 cycles of denaturation of 98 °C for 10 s, annealing at 63 °C for 30 sec, extension at 72 °C for 1 min 15 s, and a final extension of 72 °C for 10 min using an Applied Biosystems Verti 96 well thermocycler (Applied Biosystems, Foster City, CA). For glycosyl hydrolase BLNG_00015, an annealing temperature of 68 °C was used. Annealing for SBPs BLNG_00160, BLNG_00936 and BLNG_01257 was 63 °C. A total volume of 225 μl of PCR products encoding glycosyl hydrolases were gel purified and cloned into pEco-T7-cHis vectors. A volume of 100 μl of PCR products, encoding solute binding proteins, were gel-purified and cloned into pEco-T7-nGST vectors. Recombinant BL21 Star clones were confirmed via plasmid sequencing of the insert. Sequencing primers IC-1006-fwd and IC-1006-rev were used for pEco-T7-cHis vectors and primers IC-1004-fwd and IC-1004-rev were used for pEco-T7-nGST vectors. Furthermore, for plasmid inserts containing glycosyl hydrolases, intermediate primers were designed to fully sequence the glycosyl hydrolase insert. For recombinant *E. coli* BL21 Star cultures with inserts (Blng_01257-nGST, Blng_00160-nGST, Blng_00936-nGST, and Blng_01263-cHis), protein expression was conducted using 200 ml of LB supplemented with 100 ug/ml carbenicillin. For protein expression of recombinant *E. coli* BL21 Star cultures with inserts (Blng_01264-cHis and Blng_00015-cHis) 700 ml of LB with 100 ug/ml carbenicillin were used, and for recombinant *E. coli* BL21 Star cultures with insert (Blng_01753-cHis) 600 ml of LB with 100 ug/ml carbenicillin was used. Cells were grown in a shaker at 250 rpm (Innova-4000 incubation shaker; New Brunswick Scientific, Edison, NJ) until they reached an OD of 0.5 and then protein synthesis was induced with a final concentration of 0.5 mM isopropyl-β-d-thiogalactopyranoside (IPTG) overnight, with the exception of recombinant clon *E. coli* BL21 Star Blng_01263-cHis, which was induced for seven hours. *E. coli* BL21 Star cultures with plasmid inserts encoding glycosyl hydrolases were induced at 28 °C and *E. coli* BL21 Star cultures with plasmid inserts encoding solute binding proteins were induced at room temperature. Immediately following induction, recombinant *E. coli* BL21 Star cultures were centrifuged at 4000 rpm on an Eppendorf 5804 centrifuge (Hauppauge, NY) for 20 min. at 4 °C and were frozen at −80 °C. Pellets were suspended in Bugbuster protein extraction reagent (EMD chemicals) using 5 ml Bugbuster for 50 ml *E. coli* culture. For Bugbuster suspended recombinant E. coli BL21 Star cultures with GST tagged SBP inserts (Blng_00160-nGST, and Blng_00936-nGST, Blng_01257-nGST), 30 μl of DNAse I (Roche) from a 10 U/μl stock and 30 μl of lysozyme from a 100 mg/ml stock were added per 20 ml of Bugbuster suspended culture. Following inversion and a five minute incubation, the suspension was centrifuged twice for 15 min. at 13200 rpm at 4 °C, and the supernatant was applied to a 1 ml Bio-Scale Mini Profinity GST column attached to a EP-1 Econo Pump (Bio-rad, Hercules, CA). For purification of the SBPs Blng_01257-nGST, Blng_00160-nGST, Blng_00936-nGST the final step was the exchange of glutathione with PBS using 30 kDa cutoff Amicon Ultra-15 centrifugal units (Millipore, Billerica, CA). For Bugbuster suspended recombinant *E. coli* BL21 Star cultures with histidine (His) tagged glycosyl hydrolase inserts (Blng_00015-cHis, Blng_1263-cHis, Blng_1264-cHis, and Blng_01753-cHis), 60 μl of DNAse I (Roche) from a 10 U/μl stock and 120 μl of lysozyme from a 50 mg/ml stock were added per 20 ml of Bugbuster suspended culture with the exception of recombinant Blng_01753-cHis where 180 μl of lysozyme was added. Following inversion and a five minute incubation, the suspension was centrifuged for 30 min. at 13200 rpm at 4 °C, and the supernatant was applied to a 1 ml Bio-Scale Mini Profinity immobilized-metal affinity chromatography column attached to a EP-1 Econo Pump (Bio-rad, Hercules, CA. For purification of the glycosyl hydrolases the final step was the exchange of imidazole with PBS using 10 kDa cutoff Amicon Ultra-15 centrifugal units (Millipore, Billerica, CA) with the exception of (Blng_01263-cHis) where a 30 kDa cutoff Amicon Ultra-15 centrifugal unit was used (Millipore, Billerica, CA). Recombinant proteins were checked for purity and the correct size on 4–15% SDS-PAGE gels.

### Glycan array

GST-tagged family 1 SBPs were screened by the Core H of the Consortium for Functional Glycomics against Version 5.1 of the Mammalian Glycan Array to examine for glycan interaction. SBPs were diluted to a protein concentration of 200 ug/ml in binding buffer and assayed. The binding buffer composition was 20 mM Tris-HCl pH 7.4, 150 mM sodium chloride, 2 mM calcium chloride, 2 mM magnesium chloride, 0.05% Tween 20, and 1% BSA. An anti-GST Alexa 488 antibody was used for binding and detection of protein on the array. The results were expressed in relative fluorescent units (RFU). Six replicates were carried out and the highest and lowest point from each set of replicates was removed.

### Optimum enzyme conditions

To measure activity for β-galactosidases, the OD_420_ was measured using a 96 well microplate using an Eon microplate reader (BioTek) in a total assay volume of 100 μl with 1 mg/ml final concentration ONPG (2-Nitrophenyl-β-D-galactopyranoside; Sigma-Aldrich). To measure α-fucosidase activity, the OD_405_ was measured using a 96 well microplate using an Eon microplate reader (BioTek) in a total assay volume of 100 μl with 2 mg/ml final concentration CNP-fucose (2-chloro-4-nitrophenyl-α-L-fucopyranoside; Carbosynth, Berkshire, United Kingdom). To determine pH optimum for BLNG_00015, BLNG_01264, and BLNG_01753, McIlvaine buffers were prepared within the pH range of 4–8 to determine at temperature 37 °C using the above conditions. The optimum temperature for enzyme activity for BLNG_00015, BLNG_01264, and BLNG_01753 was found by incubating the purified enzyme at optimum pH in temperatures 20, 30, 37, 45, 55, and 65 °C in a 100 μl total assay volume using conditions similar as those used to determine pH optimum. For BLNG_00015, 0.001 mg of protein was used to examine both temperature and pH optimum. For BLNG_01753, 0.001 mg was used to examine pH optimum, and 0.0006 mg of protein was used to examine the temperature optimum. The time of incubation for BLNG_00015, BLNG_01264, and BLNG_01753 was 1.5 min, 6 min and 30 s respectively. For pH and temperature assays, reactions were terminated by either the addition of an equal volume of 1 M Na2CO3 or for certain assays boiling at 95 °C for 2 minutes. Reactions were all terminated using the same method for a particular enzyme assay.

### Mass spectrometry analysis of enzyme digestions

#### Enzyme Digestion

Purified recombinant enzymes were stored at −80 °C until analysis. A reduced human milk oligosaccharide (HMO) pool (1.5 μL, 5 mg/mL) was combined with 3 μL of each enzyme and 5.5 μL of 0.1 M ammonium acetate buffer. Incubation conditions were specific for each enzyme based on optimum pH and temperature values previously determined. BLNG_1264 reaction was performed at pH 7.0 and 45 °C for 1 and 3 hours using 1 mg/ml of enzyme. BLNG_00015 and BLNG_01753 incubations with HMO were performed at 55 °C for 1 hour using 3 μL 2 mg/ml of enzyme, at pH 6.0 for BLNG_00015, and 5.5 for BLNG_01753. For BLNG_1263 assay was carried out at pH 5 and 37 °C for 3 hours using 0.06 mg of enzyme using 1.5 uL of enzyme, 1.5 uL of HMO pool, and 7 uL of ammonium acetate buffer.

#### Nano-HPLC-Chip/TOF Mass Spectrometry

Analysis of the digested HMO was performed on a nano-HPLC-Chip/TOF MS system. Liquid chromatography is done on an Agilent 1200 series unit which incorporates a capillary pump for sample loading at a flow rate of 4.0 μL/min and 1 μL injection volume, and an analytical pump for analyte separation. A binary gradient was used for separation, with aqueous solvent A (3% acetonitrile:water (v/v) in 0.1% formic acid) and organic solvent B (90% acetonitrile:water (v/v) in 0.1% formic acid). Loading and separation was achieved on a microfluidic chip, which contains a 40 nL enrichment column and a 75 μL × 43 mm analytical column packed with porous graphitized carbon. The unit is coupled to an Agilent 6220 series time-of-flight mass spectrometer via chip-cube interface. Data was collected in the positive mode, and the instrument was calibrated with a dual nebulizer electrospray source and internal calibrant ions ranging from m/z 118.086 to 2721.895. The method developed for separating HMO mixtures by Wu *et al*. was used^12^.

#### Data Processing

Data was collected and analyzed using Agilent MassHunter Qualitative Analysis software, version B.03.01. The Find by Molecular Feature function was used to identify HMO compounds, and an in-house program was used for peak alignment. Specific structures were identified and assigned by matching exact mass, within 20 ppm mass error, and retention time to previously developed annotated HMO libraries^11^,^12^. Absolute abundances as extracted in ion counts were directly correlated to the abundance of that compound. To calculate the consumption of specific structures, an undigested HMO pool was run along with the digested HMO samples. The abundance of structures extracted from the undigested pool were compared to those from the digested pool using equation (2):


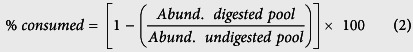


Twenty-two distinctive structures were identified. If there were two closely eluting peaks and the isomer abundances could not be integrated separately, those peak abundances were summed and the two isomers grouped together.

## Additional Information

**How to cite this article**: Garrido, D. *et al*. A novel gene cluster allows preferential utilization of fucosylated milk oligosaccharides in *Bifidobacterium longum* subsp. *longum* SC596. *Sci. Rep.* **6**, 35045; doi: 10.1038/srep35045 (2016).

## Supplementary Material

Supplementary Information

## Figures and Tables

**Table 1:** Growth of infant-associated *B. longum* strains on different complex sugars associated with human milk.

Complex Milk Sugars									
Strains	HMO	LNT	LNnT	2FL	3FL	3SL	6SL	Mucin	LAC
SC91	++^a^	+++	+	−	−	−	−	+	+++
SC116	++	+++	+	−	−	−	−	+	+++
SC156	++	+++	++	−	−	−	−	+	+++
SC215	++	+++	−	−	−	−	−	+	+++
SC249	−	−	−	−	−	−	−	−	−
SC280	++	+++	+	−	−	−	−	+	+++
SC513	+	+++	+	−	−	−	−	+	+++
SC536	++	++	−	−	−	−	−	−	+++
SC558	++	+++	+++	−	+	−	−	+	+++
SC592	++	+++	+	−	−	−	−	−	+++
SC596	++	+++	++	+++	+++	−	−	+	+++
SC618	++	+++	++	−	−	−	−	−	+++
SC630	++	+++	++	−	−	−	−	−	+++
SC657	+	+++	−	−	−	−	−	−	+++
SC662	+	+++	−	−	−	−	−	−	+++
SC664	+	+++	+++	−	−	−	−	+	+++
SC697	++	+++	−	−	−	−	−	+	+++
ATCC 15697	+++	+++	+++	+++	+++	++	+++	−	+++
PRL2010	nd^b^	nd	nd	nd	nd	nd	nd	++	++
JCM10602	−	−	−	−	−	−	−	−	++

^a^Level of growth was classified as follows: −, negative (maximum OD600 < 0.2); +, low (OD600 from 0.2 to 0.5); ++, moderate (OD600 from 0.5 to 0.8); +++, high (OD600 > 0.8).

^b^nd: not determined

Control strains; *B. infantis* ATCC15697, *B. bifidum* PRL2010 and *B. animalis* subsp*. lactis* JCM10602 are also shown.

**Table 2:** Binding affinities of *B. longum* SC596 encoded Solute Binding Proteins for mammalian glycans.

*B. longum* SC596 SBP gene and glycan structure bound	Average RFU^b^
**BLNG_00160**	
** Galβ1-3Gal**β1-4GlcNAcβ-Sp8	4654
** Galβ1-3GalNAcβ1**-4Galβ1-4Glcβ-Sp8	3430
** Galβ1-3GlcNAcβ1**-3Galβ1-4GlcNAcβ1-6(**Galβ1-3GlcNAcβ1**-3Galβ1-4GlcNAβ1-2)Mana1-6 (**Galβ1-3GlcNAcβ1**-3Galβ1-4GlcNAcβ1-2Mana1-3)Manβ1-4GlcNAcβ1-4(Fuca1-6)GlcNAcβ-Sp24	3351
Neu5Acα2-6(**Galβ1-3**)GlcNAcβ1-4Galβ1-4Glcβ-Sp10	2706
** Galβ1-3(6S)GlcNAcβ**-Sp8	2646
** Galβ1-3GalNAcβ1**-3Gal-Sp21	2528
** Galβ1-3GlcNAcβ1**-3Galβ1-4GlcNAcβ1-3Galβ1-4GlcNAcβ1-6(**Galβ1-3GlcNAcβ1**-3Galβ1-4GlcNAcβ1-3Galβ1-4GlcNAβ1-2)Mana1-6(**Galβ1-3GlcNAcβ1**-3Galβ1-4GlcNAcβ1-3Galβ1-4GlcNAcβ1-2Mana1-3)Manβ1-4GlcNAcβ1-4(Fuca1-6)GlcNAcβ-Sp24	2513
** Galβ1-3GalNAcβ**-Sp8	2338
** Galβ1-3GlcNAcβ1**-3Galβ1-4GlcNAcβ1-6(**Galβ1-3GlcNAcβ1**-3)Galβ1-4Glcβ-Sp0	2212
** Galβ1-3GlcNAcβ1**-3Galβ1-4GlcNAcβ1-2Mana1-6(Galβ1-3GlcNAcβ1-3Galβ1-4GlcNAcβ1-2Mana1-3)Manβ1-4GlcNAcβ1-4GlcNAc-Sp25	2079
** Galβ1-3GlcNAcβ1**-3Galβ1-4GlcNAcβ-Sp0	2029
** Galβ1-3GlcNAcβ1**-3Galβ1-4Glcβ-Sp10	1784
** Galβ1-3Galβ**-Sp8	1508
** Galβ1-3GlcNAcβ**-Sp0	969
**BLNG_00936**	
** Galβ1-3GalNAcβ**-Sp8	303
** Galβ1-3Galβ1**-4GlcNAcβ-Sp8	126
**BLNG_01257**	
** Fuca1-2Galβ**-Sp8	2161
** Fuca1-2Galβ1**-4Glcβ-Sp0	360

In bold are shown common motifs recognized by any SBP. Spacers (Sp) are repeats of CH = CH units to a glass slide in the Glycan Array, and can contain repeats from 0 to 25 units. RFU: Relative fluorescence Units.
