# Supplementary material for: A novel gene cluster allows preferential utilization of fucosylated milk oligosaccharides in Bifidobacterium longum subsp. longum SC596
Source: Sci Rep. 2016 Oct 19;6:35045. doi: 10.1038/srep35045 (PMC5069460; doi:10.1038/srep35045)
Supplement: Supplementary Information [file srep35045-s1.pdf]

A novel gene cluster allows preferential utilization of fucosylated milk oligosaccharides in *Bifidobacterium longum* subsp. *longum* SC596.

Daniel Garrido[2,4,6], Santiago Ruiz-Moyano[2,4], Nina Kirmiz[2,4], Jasmine C. C. Davis[3,4], Sarah Totten[3,4], Danielle G. Lemay[4,5], Juan A. Ugalde[7], J. Bruce German[1,4], Carlito B. Lebrilla[3,4], and David A. Mills[1,2,4]*.

SUPPLEMENTAL MATERIAL

This article contains 9 Supplementary Tables and 6 Supplementary Figures.

LEGEND TO SUPPLEMENTARY FIGURES

FIGURE S1: Evolutionary relationship of *B. longum* strains in this study estimated by MLST analysis. The tree is drawn to scale, with branch lengths in the same units (number of base substitutions per site) as those of the evolutionary distances used to infer the phylogenetic tree. The evolutionary history was inferred using the Minimum Evolution method, followed by 1000 bootstrap replicates.

FIGURE S2: Growth of *B. longum* isolates on mMRS medium supplemented with A: 2% HMO; B: 2% LNT; C: 2% LNnT; D: 2% 3SL; E: 2% 6SL; F: 1% mucin. Growth was measured as OD of the media at 600 nm. Fermentations were carried out in triplicate; controls consisted of inoculated medium lacking of substrates and un-inoculated medium containing substrates which was also used as blank for OD measurements.

FIGURE S3: Principal component analysis (PCA) of the maximum optical density values (600 nm) of *B. longum* strains grown on HMO, LNT, LNnT, 2FL, 3FL, 3SL, 6SL and mucin. Negative control, *B. animalis* (red) and positive control *B. infantis* ATCC 15697 (green). Figure S3A represents variables (oligosaccharides substrates) and Figure S3B bifidobacteria strains.

FIGURE S4: Phylogenomic reconstruction based on 275 genes shared between all the studied *B. longum* genomes (total alignment of 202,423 nucleotides);

FIGURE S5: Venn diagram representing unique and shared genes between *B. longum* SC596 and DJO10A.

FIGURE S6: *B. longum* SC596 additional gene clusters related to HMO consumption. Colors are indicative of primary function of each respective gene, transcriptional regulators (black), oligosaccharide transport (blue) and carbohydrate feeder pathways (green).

TABLE S1: List of *Bifidobacterium* strains used in this study.

Code	Species	Additional strain information[a]	Source
SC91	*B. longum subsp. longum*	This study	Infant feces
SC116	*B. longum subsp. longum*	This study	Infant feces
SC156	*B. longum subsp. longum*	This study	Infant feces
SC215	*B. longum subsp. longum*	This study	Infant feces
SC249	*B. longum subsp. longum*	This study	Infant feces
SC280	*B. longum subsp. longum*	This study	Infant feces
SC513	*B. longum subsp. longum*	This study	Infant feces
SC536	*B. longum subsp. longum*	This study	Infant feces
SC558	*B. longum subsp. longum*	This study	Infant feces
SC592	*B. longum subsp. longum*	This study	Infant feces
SC596	*B. longum subsp. longum*	This study	Infant feces
SC618	*B. longum subsp. longum*	This study	Infant feces
SC630	*B. longum subsp. longum*	This study	Infant feces
SC657	*B. longum subsp. longum*	This study	Infant feces
SC662	*B. longum subsp. longum*	This study	Infant feces
SC697	*B. longum subsp. longum*	This study	Infant feces
DJO10A	*B. longum subsp. longum*	Gene bank: NC_010816	Adult feces
KACC91563	*B. longum subsp. longum*	Gene bank: NC_017221	Neonate feces
NCC2705	*B. longum subsp. longum*	Gene bank: NC_004307	Infant feces
BBMN68	*B. longum subsp. longum*	Gene bank: NC_014656	Adult feces
JCM1217	*B. longum subsp. longum*	ATCC15707; DSMZ20219	Adult feces
157F	*B. longum subsp. longum*	Gene bank: NC_015052	Infant feces
F8	*B. longum subsp. longum*	Gene bank: NC_021008	Human feces
BXY01	*B. longum subsp. longum*	Gene bank: CP008885	-
JDM301	*B. longum subsp. longum*	Gene bank: NC_014169	Probiotic product
ATCC15697	*B. longum subsp. infantis*	JCM1222; DSMZ20088	Infant feces
PRL2010	*B. bifidum*	Turroni et al. 2010	Infant feces
JCM10602	*B. animalis subsp. lactis*	DSMZ 10140	Dairy product

[a]The original strain numbers are also noted, if known. JCM, Japan Collection of Microorganisms, ATCC, American Type Culture Collection; DSMZ, German Collection of Microorganisms and Cell Culture.

Table S2: Structures and masses of human milk oligosaccharides analyzed in this study.

HMO type		Name	Abbreviation of designation	Mass *(m/z)*	Structure	DP
Neutral	non fucosylated	Lacto-*N*-tetraose	LNT	709	Galβ1-4GlcNacβ1-3Galβ1-4Glc	4
		Lacto-*N*-neotetraose	LNnT	709	Galβ1-4GlcNacβ1-3Galβ1-4Glc	4
		Lacto-*N*-hexaose	LNH	1074	Galβ1-3GlcNacβ1-3[Galβ1-4GlcNacβ1-6]Galβ1-4Glc	6
		Lacto-*N*-neohexaose	LNnH	1074	Galβ1-4GlcNacβ1-3[Galβ1-4GlcNacβ1-6]Galβ1-4Glc	6
		para-Lacto-*N*-hexaose	p-LNH	1074	Galβ1-3GlcNacβ1-3Galβ1-4GlcNacβ1-3Galβ1-4Glc	6
	fucosylated	2’-Fucosyllactose	2FL	490	Fucα1-2Galβ1-4Glc	3
		Lactodifucotetraose	LDFT	636	Fucα1-2Galβ1-[Fucα1-3]4Glc	4
		Lacto-*N*-fucopentaose	LNFPI	855	Fucα1-2Galβ1-3GlcNacβ1-3Galβ1-4Glc	5
			LNFPII	855	Galβ1-3[Fucα1-4]GlcNacβ1-3Galβ1-4Glc	5
			LNFPIII	855	Galβ1-3[Fucα1-3]GlcNacβ1-3Galβ1-4Glc	5
		Difucosyllacto-*N*-tetraose	3_1_2_0	1074	Fucα1-2Galβ1-4[Fucα1-4]GlcNacβ1-3Galβ1-4Glc/ Galβ1-4[Fucα1-4]GlcNacβ1-3Galβ1-4[Fucα1-3]Glc	6
		Monofucosylacto-*N*-hexaose	MFLNHI	1221	Fucα1-2Galβ1-3GlcNacβ1-3[Galβ1-4GlcNacβ1-6]Galβ1-4Glc	7
			MFLNHIII	1221	Galβ1-3GlcNacβ1-3[Galβ1-4[Fucα1-3]GlcNacβ1-6]Galβ1-4Glc	7
			4120a	1221	Galβ1-3[Fucα1-4]GlcNacβ1-3[Galβ1-4GlcNacβ1-6]Galβ1-4Glc	7
		Monofucosylparalacto-*N*-hexaose	IFLNH I	1221	Fucα1-2Galβ1-4GlcNacβ1-3Galβ1-4GlcNacβ1-3Galβ1-4Glc	7
			IFLNH III	1221	Galβ1-4GlcNacβ1-3Galβ1-4[Fucα1-3]GlcNacβ1-3Galβ1-4Glc	7
		Monofucosylparalacto-*N*-hexaose	MFpLNHIV	1221	Galβ1-3GlcNacβ1-3Galβ1-4[Fucα1-3]GlcNacβ1-3Galβ1-4Glc	7
		Difucosyllacto-*N*-hexaose	DFLNHa	1366	Fucα1-2Galβ1-3GlcNacβ1-3[Galβ1-4[Fucα1-4]GlcNacβ1-6]Galβ1-4Glc	8
		Trifucosyllacto-*N*-hexaose	DFLNHb	1366	Galβ1-3[Fucα1-4]GlcNacβ1-3[Galβ1-4[Fucα1-3]GlcNacβ1-6]Galβ1-4Glc	8
			DFLNHc	1366	Fucα1-2Galβ1-3[Fucα1-4]GlcNacβ1-3[Galβ1-4GlcNacβ1-6]Galβ1-4Glc	8
			DFpLNHII	1366	Galβ1-3[Fucα1-4]GlcNacβ1-3Galβ1-4[Fucα1-3]GlcNacβ1-3Galβ1-4Glc	8
			TFLNH	1513	Fucα1-2Galβ1-3GlcNacβ1-3[Fucα1-2Galβ1-3GlcNacβ1-3Galβ1-4GlcNacβ1-6]Galβ1-4Glc	9
		Monofucosyllacto-*N*-octaose	5130a	1585	Galβ1-3GlcNacβ1-3Gal[Galβ1-3GlcNacβ1-3Galβ1-4[Fucα1-3]GlcNacβ1-6]Galβ1-4Glc	9
		Difucosyllacto-*N*-octaose	5130b	1585	Galβ1-3[Fucα1-4]GlcNacβ1-3Gal[Galβ1-4GlcNacβ1-3Galβ1-4GlcNacβ1-6]Galβ1-4Glc	9
			5130c	1585	Fucα1-2Galβ1-3GlcNacβ1-3Gal[Galβ1-4GlcNacβ1-3Galβ1-4GlcNacβ1-6]Galβ1-4Glc	9
			F-LNO	1585	Galβ1-3GlcNacβ1-3Gal[Galβ1-4GlcNacβ1-3Galβ1-4[Fucα1-3]GlcNacβ1-6]Galβ1-4Glc	9
			DFLNO I	1731	Galβ1-3GlcNacβ1-3Gal[Galβ1-4[Fucα1-3]GlcNacβ1-3Galβ1-4[Fucα1-3]GlcNacβ1-6]Galβ1-4Glc	10
		Difucosyllacto-*N*-neooctaose	DFLNnO II	1731	Galβ1-4[Fucα1-3]GlcNacβ1-3Gal[Galβ1-3GlcNacβ1-3Galβ1-4[Fucα1-3]GlcNacβ1-6]Galβ1-4Glc	10
		Difucosyllacto-*N*-neooctaose	DFLNnO I/ DFLNO II	1731	Galβ1-4GlcNacβ1-3Gal[Galβ1-3[Fucα1-4]GlcNacβ1-3Galβ1-4[Fucα1-3]GlcNacβ1-6]Galβ1-4Glc/ Galβ1-3[Fucα1-4]GlcNacβ1-3Gal[Galβ1-4GlcNacβ1-3Galβ1-4[Fucα1-3]GlcNacβ1-6]Galβ1-4Glc	10
		Isomer Difucosyllacto-*N*-octaose	5230a	1731	Galβ1-3GlcNacβ1-3Gal[Fucα1-2Galβ1-3GlcNacβ1-3Galβ1-4[Fucα1-3]GlcNacβ1-6]Galβ1-4Glc	10
		Difucosyllacto-*N*-octaose				
		Trifucosyllacto-*N*-octaose	5330a	1877	Fucα1-2-Galβ1-3[Fucα1-4]GlcNacβ1-3Gal[Fucα1-2Galβ1-3GlcNacβ1-3Galβ1-4GlcNacβ1-6]Galβ1-4Glc	11
Acidic	Sialylated	3-Sialylactose	3SL	635	NeuAcα2-3Galβ1-4Glc	3
		Monosialyllacto-*N*-tetraose	LSTa	1000	NeuAcα2-3Galβ1-3GlcNacβ1-3Galβ1-4Glc	5
		Monosialyllacto-*N*-neotetraose	LSTc	1000	NeuAcα2-6Galβ1-4GlcNacβ1-3Galβ1-4Glc	5
		Monosialyllacto-*N*-hexaose	S-LNH	1365	Galβ1-3GlcNacβ1-3[[NeuAcα2-6]Galβ1-4GlcNacβ1-6]Galβ1-4Glc	5

		Monosialyllacto-*N*-neohexaose	4021a	1365	Galβ1-3[NeuAcα2-6]GlcNacβ1-3[Galβ1-4GlcNacβ1-6]Galβ1-4Glc	5
			S-LNnHII	1365	[NeuAcα2-6]Galβ1-4GlcNacβ1-3[Galβ1-4GlcNacβ1-6]Galβ1-4Glc	7
		unknown	4_2_1_1.b	1511	Not defined	8

Table S3: Glycomic potential of *B. longum* SC596

Function ID	Name	1-6B	2-2B	35B	44B	AGR2 137	D2957	DJO1 0A	E18	NCC2 705	157F-NC	ATCC 15697	ATCC 55813	BBM N68	CCUG 52486	CMCC P0001	F8	JCM1 217	JDM3 01	KACC 91563	SC59 6
EC:3.2.1.-	Hydrolases. Glycosylases. Glycosidases	2	2	2	2	2	2	2	2	2	2	2	2	2	2	2	2	2	2	2	2
EC:3.2.1.1	Alpha-amylase.	1	0	0	1	1	1	1	0	0	1	0	1	1	1	1	0	1	1	1	1
EC:3.2.1.10	Oligo-1,6-glucosidase.	0	2	1	0	3	1	1	1	1	2	1	0	2	2	1	2	1	1	2	2
EC:3.2.1.156	Oligosaccharide reducing-end xylanase.	0	0	0	0	0	0	0	0	0	0	0	0	0	0	1	0	0	1	0	0
EC:3.2.1.177	Alpha-D-xyloside xylohydrolase.	1	1	1	1	0	1	1	1	0	1	0	1	1	1	1	1	1	1	1	1
EC:3.2.1.18	Exo-alpha-sialidase.	0	0	0	0	0	0	0	0	0	0	2	0	0	0	0	0	0	0	0	0
EC:3.2.1.185	Non-reducing end beta-L-arabinofuranosidase.	1	1	1	1	0	1	0	1	1	1	0	1	1	1	0	1	1	0	1	1
EC:3.2.1.187	(Ara-f)(3)-Hyp beta-L-arabinobiosidase.	1	1	1	1	0	1	0	1	1	1	0	1	1	1	0	1	1	0	1	1
EC:3.2.1.20	Alpha-glucosidase.	1	2	2	1	1	2	2	2	1	1	1	2	2	1	2	2	2	2	2	2
EC:3.2.1.21	Beta-glucosidase.	2	4	4	4	6	3	2	3	1	4	2	4	4	3	3	5	3	3	3	5
EC:3.2.1.22	Alpha-galactosidase.	1	1	1	1	1	1	1	1	1	1	1	1	1	1	1	1	1	1	1	1
EC:3.2.1.23	Beta-galactosidase.	3	3	3	3	4	3	3	3	3	4	4	4	3	3	4	3	3	4	3	3
EC:3.2.1.24	Alpha-mannosidase.	0	1	0	2	2	0	3	2	3	2	2	2	2	0	3	2	0	3	0	2
EC:3.2.1.26	Beta-fructofuranosidase.	1	1	1	1	1	1	1	1	1	1	1	1	1	1	1	1	1	1	1	1
EC:3.2.1.31	Beta-glucuronidase.	1	0	0	1	1	1	1	1	0	0	0	0	0	1	1	1	1	1	1	1
EC:3.2.1.37	Xylan 1,4-beta-xylosidase.	1	0	0	1	1	0	1	0	0	1	0	0	0	1	1	1	0	1	1	1
EC:3.2.1.41	Pullulanase.	0	0	0	0	1	0	0	0	0	0	0	0	0	0	1	0	0	1	0	0
EC:3.2.1.45	Glucosylceramidase.	0	0	0	0	1	1	0	1	0	0	0	0	1	0	0	0	1	0	0	0
EC:3.2.1.51	Alpha-L-fucosidase.	0	0	0	0	1	0	0	0	0	0	4	0	0	0	2	0	0	2	0	2
EC:3.2.1.52	Beta-N-acetylhexosaminidase.	1	1	1	1	1	1	1	1	1	1	1	1	1	1	1	0	1	1	1	1
EC:3.2.1.55	Non-reducing end alpha-L-arabinofuranosidase.	4	4	4	4	1	4	4	4	4	4	0	4	3	3	2	3	4	2	4	4
EC:3.2.1.58	Glucan 1,3-beta-glucosidase.	1	1	1	0	0	1	1	1	1	1	1	1	1	1	1	1	1	1	1	1

EC:3.2.1.89	Arabinogalactan endo-beta-1,4-galactanase.	1	1	1	1	1	0	1	1	1	1	0	0	0	0	1	1	0	1	0	1
EC:3.2.1.97	Endo-alpha-N-acetylgalactosaminidase.	1	1	1	1	1	1	1	1	1	1	0	1	1	1	0	0	1	0	1	1
EC:3.2.1.99	Arabinan endo-1,5-alpha-L-arabinosidase.	2	2	2	2	0	0	2	0	2	0	0	2	0	2	0	1	0	0	0	2
COG1653	ABC-type sugar transport system, periplasmic component	11	12	12	11	12	9	14	12	10	12	20	12	11	11	20	15	9	20	14	17

Table S4: General features of RNA-seq experiments

Sample List	Number of reads	Counted fragments genes	Counted fragments intergenic	Uncounted fragments	% uncounted	% reads align genes	% Intergenic	%16S	%23S	%5S
LAC-A	9888033	7594423	1131340	1162270	11.75	76.80	11.44	0.16	1.04	0.00
LAC-B	5654963	4243518	696941	714504	12.63	75.04	12.32	0.25	1.71	0.00
HMOearly-A	7482193	5996053	655133	831007	11.11	80.14	8.76	0.27	0.78	0.00
HMOearly-B	7575569	6010964	652037	912568	12.05	79.35	8.61	0.04	0.35	0.00
HMOmid1-A	6234472	5003960	515462	715050	11.47	80.26	8.27	0.03	0.34	0.01
HMOmid1-B	5394738	4293100	471296	630342	11.68	79.58	8.74	0.06	0.67	0.01
HMOmid2-A	6428553	4826995	599781	1001777	15.58	75.09	9.33	0.11	1.15	0.00
HMOmid2-B	5124926	4016256	555846	552824	10.79	78.37	10.85	0.42	4.21	0.00
HMOlate-A	5611251	3963333	912225	735693	13.11	70.63	16.26	0.14	1.86	0.00
HMOlate-B	4118311	3104249	561952	452110	10.98	75.38	13.65	0.03	0.42	0.00
LNT-A	8932952	6832417	1192980	907555	10.16	76.49	13.35	0.11	0.90	0.01
LNT-B	9145050	7230021	1076957	838072	9.16	79.06	11.78	0.02	0.70	0.01
LNnT-A	10064954	8377587	834361	853006	8.48	83.24	8.29	1.00	9.27	0.02
LNnT-B	12872241	10630312	1163154	1078775	8.38	82.58	9.04	0.71	4.24	0.02
2FL-A	8052846	5836574	1172461	1043811	12.96	72.48	14.56	0.03	1.46	0.00
2FL-B	23080453	17134484	3079955	2866014	12.42	74.24	13.34	0.04	1.89	0.01
3FL-A	10734330	7607429	1787026	1339875	12.48	70.87	16.65	0.05	2.76	0.00
3FL-B	17245753	12111408	2799942	2334403	13.54	70.23	16.24	0.04	2.72	0.00
Total reads	163641588	124813083								
Average B. longum	9091199	6934060	1103269	1053870	11.60	76.66	11.75	0.19	2.03	0.01
SD	4728266	3471806	749151	609837	1.81	3.98	2.95	0.27	2.17	0.01

[a] Total reads aligning to intergenic regions, identified in both genomes.

[b] Total reads not aligned to any genic or intergenic region.

Table S5: Transcriptomics of HMO utilization in *B. longum* SC596: Differentially regulated genes in *B. longum* SC596, relative to lactose

Treatment	Number Differentially Expressed genes
2'FL	1070
3'FL	1308
LNT	1071
LNnT	1299
HMO Early	1353
HMO Mid1	1061
HMO Mid2	1239
HMO Late	1206

Table S6: Transcriptomics of HMO utilization in *B. longum* SC596: Enriched annotations in *B. longum* SC596 genes up-regulated relative to lactose

Type	ANNOT_ID	ANNOT_DESC	HMOe	HMOm1	HMOm2	HMOl	LNT	LNnT	2FL	3FL
COG	COG3507	Beta-xylosidase	NS	NS	0.013	NS	NS	NS	NS	NS
COG	COG1136	ABC-type antimicrobial peptide transport system, ATPase component	NS	NS	NS	NS	0.042	NS	NS	NS
KEGG	MAP00030	Pentose phosphate pathway	NS	NS	0.008	NS	NS	NS	NS	NS
KEGG	MAP00970	Aminoacyl-tRNA biosynthesis	0.003	0.061	NS	NS	NS	NS	NS	NS
KEGG	MAP03010	Ribosome	0	0	NS	NS	NS	NS	NS	NS
SRI	TRNA-CHARGING-PWY	tRNA charging	0	NS	NS	NS	NS	NS	0	0
SRI	PWY-6717	(1,4)-β-xylan degradation	0.003	NS	0.013	0.023	NS	NS	NS	0.04
GO	GO:0008643	carbohydrate transport	NS	NS	NS	0.014	NS	NS	NS	NS
GO	GO:0006950	response to stress	NS	NS	NS	0.016	NS	NS	NS	NS
GO	GO:0005840	ribosome	0.001	0.001	NS	NS	NS	NS	NS	NS
GO	GO:0019843	rRNA binding	0	0.004	NS	NS	NS	0.018	NS	NS

Type	ANNOT_ID	ANNOT_DESC	HMOe	HMOm1	HMOm2	HMOl	LNT	LNnT	2FL	3FL
GO	GO:0003735	structural constituent of ribosome	0	0	NS	NS	NS	NS	NS	NS
GO	GO:0006200	ATP catabolic process	NS	0.005	NS	NS	NS	NS	NS	NS
GO	GO:0006412	translation	0	0.001	NS	NS	NS	NS	NS	NS
GO	GO:0005524	ATP binding	NS	0.047	NS	NS	NS	NS	NS	NS

Table S7: Transcriptomics of HMO utilization in *B. longum* SC596: Enriched annotations in *B. longum* down-regulated relative to lactose

TYPE	ANNOT_ID	ANNOT_DESC	HMOe	HMOm1	HMOm2	HMOl	LNT	LNnT	2FL	3FL
COG	COG1609	Transcriptional regulators	0.003	NS	NS	NS	0.017	NS	NS	NS
SRI	PWY0-781	aspartate superpathway	NS	NS	NS	NS	0.028	NS	NS	NS

The table lists p-values that are adjusted for multiple hypothesis testing. Green highlight (adjusted p <= 0.05) is significant, “NS” is non-significant, and others are marginally significant (unadjusted p <= 0.05). Only terms with at least one significant condition are listed.

Table S8: Putative genes associated to HMO consumption in *B. longum* SC596 (Related to Figure 5).

Feature ID	Gene	Gene description
BLNG_00936	984	ABC-type sugar transport system, periplasmic component
BLNG_00160	1.317	ABC-type sugar transport system, periplasmic component
BLNG_01262	438	Fucose dissimilation pathway protein FucU(EC:5.1.3.-)
BLNG_01258	1.278	L-alanine-DL-glutamate epimerase and related enzymes of enolase superfamily
BLNG_01263	1.437	Alpha-L-fucosidase(EC:3.2.1.51)
BLNG_01259	792	Dehydrogenases with different specificities (related to short-chain alcohol dehydrogenases)(
BLNG_01260	822	Predicted metal-dependent hydrolase of the TIM-barrel fold
BLNG_01261	897	Dihydrodipicolinate synthase/N-acetylneuraminate lyase(EC:4.2.1.52)
BLNG_01264	2.352	hypothetical protein
BLNG_01257	1.383	ABC-type sugar transport system, periplasmic component
BLNG_01255	876	ABC-type sugar transport systems, permease components
BLNG_01256	978	ABC-type sugar transport system, permease component
BLNG_00014	1.488	sugar (Glycoside-Pentoside-Hexuronide) transporter
BLNG_00196	1.014	UDP-glucose-4-epimerase(EC:5.1.3.2)
BLNG_01135	1.251	galactose-1-phosphate uridylyltransferase, family 1(EC:2.7.7.12)
BLNG_01136	1.251	galactokinase(EC:2.7.1.6)
BLNG_00166	1.023	UDP-glucose-4-epimerase(EC:5.1.3.2)
BLNG_01475	1.677	phosphoglucomutase, alpha-D-glucose phosphate-specific(EC:5.4.2.2)
BLNG_01345	1.128	ABC-type sugar transport systems, ATPase components
BLNG_00935	1.542	ABC-type sugar transport system, ATPase component(EC:3.6.3.17)
BLNG_00933	1.023	Ribose/xylose/arabinose/galactoside ABC-type transport systems, permease components
BLNG_00934	1.071	Ribose/xylose/arabinose/galactoside ABC-type transport systems, permease components
BLNG_00460	915	Transcriptional regulator/sugar kinase(EC:2.7.1.2)
BLNG_00163	2.256	1,3-beta-galactosyl-N-acetylhexosamine phosphorylase
BLNG_00164	1.080	Putative homoserine kinase type II (protein kinase fold)
BLNG_00165	1.548	Galactose-1-phosphate uridyltransferase(EC:2.7.7.12)
BLNG_00457	1.284	N-acetylglucosamine-6-phosphate deacetylase(EC:3.5.1.25)
BLNG_00458	813	glucosamine-6-phosphate isomerase(EC:3.5.99.6)
BLNG_00161	972	ABC-type sugar transport systems, permease components
BLNG_00162	951	ABC-type sugar transport system, permease component
BLNG_00911	2.136	N-acetyl-beta-hexosaminidase
BLNG_00459	1.125	Transcriptional regulator/sugar kinase
BLNG_01753	2.076	Beta-galactosidase(EC:3.2.1.23)

Table S9: MLST genes and primers used in this study.

Gene	PCR primer (5´-3´)*,a	Expected Amplicon size (bp)	Anneling Temp. (ºC)
clpC	GAG TAC CGC AAG TAC ATC GAG	748	63
	CAT CCT CAT CGT CGA ACA GGA AC		
purF	CAT TCG AAC TCC GAC ACC GA	977	62
	GTG GGG TAG TCG CCG TTG		
gyrB	AGC TGC ACG CBG GCG GCA AGT TCG	811	66
	GTT GCC GAG CTT GGT CTT GGT CTG		
fusA	ATC GGC ATC ATG GCY CAC ATY GAT	784	66
	CCA GCA TCG GCT GMA CRC CCT T		
Iles	ATC CCG CGY TAC CAG ACS ATG	789	66
	CGG TGT CGA CGT AGT CGG CG		
rplB	GGA CAA GGA CGG CRT SCC SGC CAA	498	67
	ACG ACC RCC GTG CGG GTG RTC GAC		
rpoB	GGC GAG CTG ATC CAG AAC CA	1057	62
	GCA TCC TCG TAG TTG TAS CC		

*= Upper sequence, forward primer; Lower sequence, reverse primer.

[a] In the primer sequence R indicates (A/G), S (C/G), Y (C/T).

SBP primers used for cloning (5´-3´)

Blng_00160F TTTGTACAAAAAAGCCGGCGACACGAAGTCCGGCAG
Blng_00160R TTTGTACAAGAAACTCCTTGACGGACAGACCGAGGT
Blng_00936F TTTGTACAAAAAAGGTGGCAGCTCGGACTCCGGCAAG
Blng_00936R TTTGTACAAGAAAGTAGGCGCGGGTGTTGTTGTCC
Blng_01257F TTGTACAAAAAAAAGAGCGATGTGACCGCGCAGGAC
Blng_01257R TTTGTACAAGAAAGTCAGCGTCGGTGGTGACCTTGAA

Glycosyl hydrolases primers used for cloning (5´-3´)

Blng_00015F TTTGTACAAAAAAGCAGGCACCATGACAGACGTCACACATGTCG
Blng_00015R TTTGTACAAGAAAGCTGGGTTGATCAGCTCGAGGTCGACATCG
Blng_01753F TTTGTACAAAAAAGCAGGCACCATGGAACGTAAAGAGTTCAAGT
Blng_01753R TTTGTACAAGAAAGCTGGGTTCAGCTTGACGACAAGTACGCCG
Blng_01263F TTTGTACAAAAAAGCAGGCACCATGAGCAATCCAACAAATGATG
Blng_01263R TTTGTACAAGAAAGCTGGGTTTATCCGCACCGCAGCCGCACGG
Blng_01264F TTTGTACAAAAAAGCAGGCACCATGAAACTCACATTCGATGGAA
Blng_01264R TTTGTACAAGAAAGCTGGGTTACGCCGGATGGTTCCCTTGAAT

Figure S1

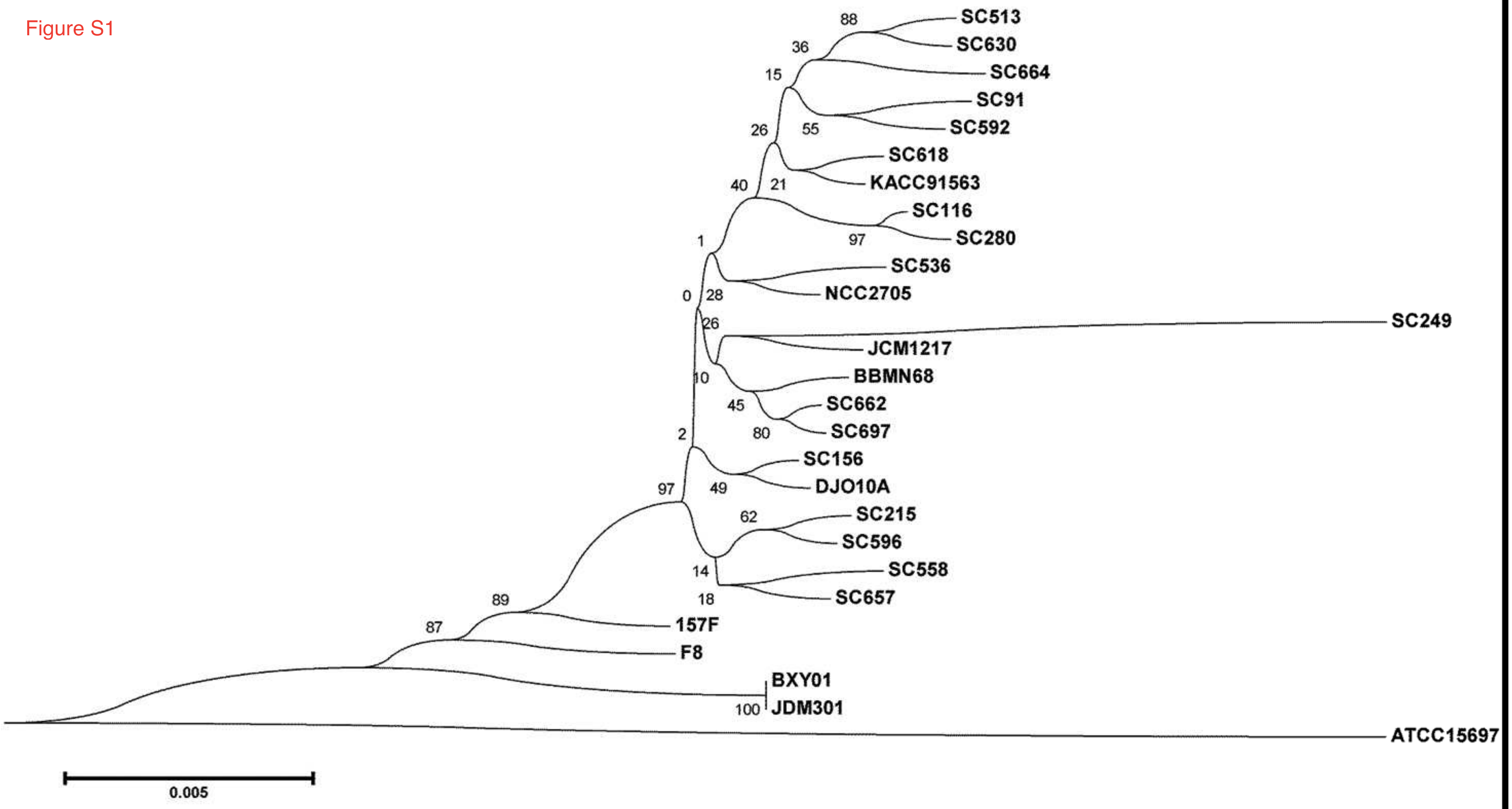

SC513
SC630
SC664
SC91
SC592
SC618
KACC91563
SC116
SC280
SC536
NCC2705
SC249
JCM1217
BBMN68
SC662
SC697
SC156
DJO10A
SC215
SC596
SC558
SC657
157F
F8
BXY01
JDM301
ATCC15697
88
36
15
55
26
21
40
97
1
0
28
26
10
45
80
2
97
49
62
14
18
89
87
100
0.005

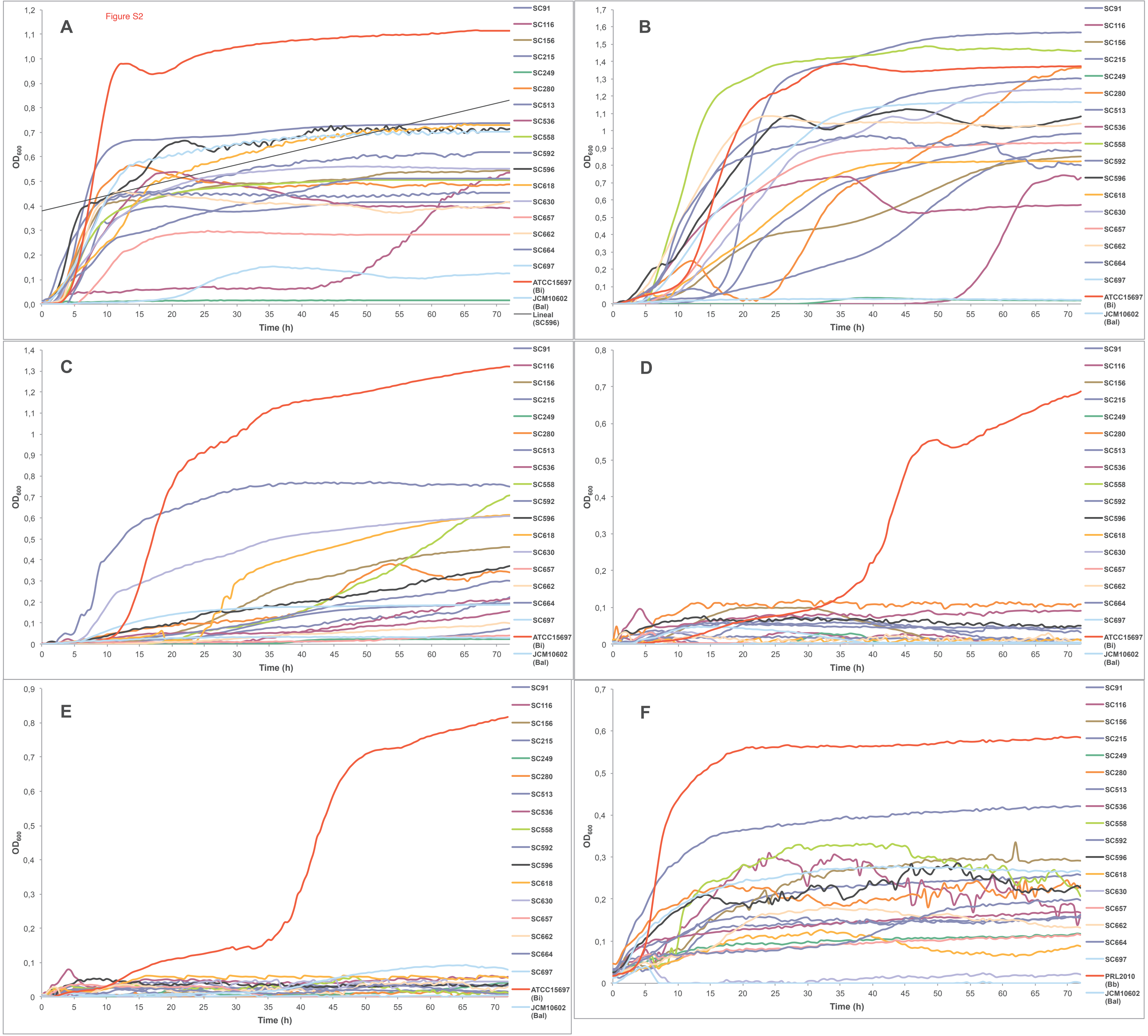
A
B
C
D
E
F
OD600
Time (h)
SC91
SC116
SC156
SC215
SC249
SC280
SC513
SC536
SC558
SC592
SC596
SC618
SC630
SC657
SC662
SC664
SC697
ATCC15697 (Bi)
JCM10602 (Bal)
Lineal (SC596)
PRL2010 (Bb)

Figure S2

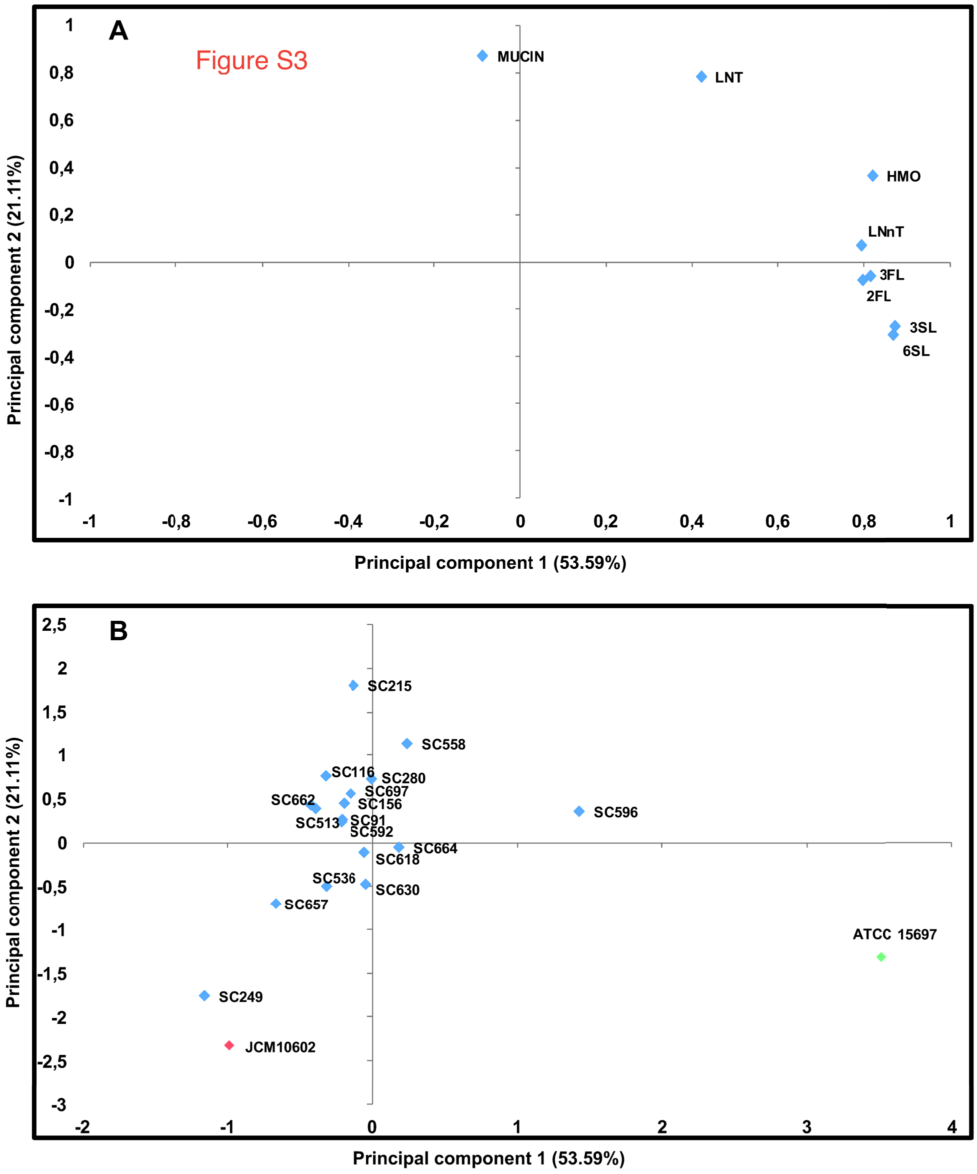
A
Figure S3
MUCIN
LNT
HMO
LNnT
3FL
2FL
3SL
6SL
Principal component 2 (21.11%)
Principal component 1 (53.59%)
B
SC215
SC558
SC116
SC280
SC697
SC662
SC156
SC513
SC91
SC592
SC596
SC664
SC618
SC536
SC630
SC657
ATCC 15697
SC249
JCM10602
Principal component 2 (21.11%)
Principal component 1 (53.59%)

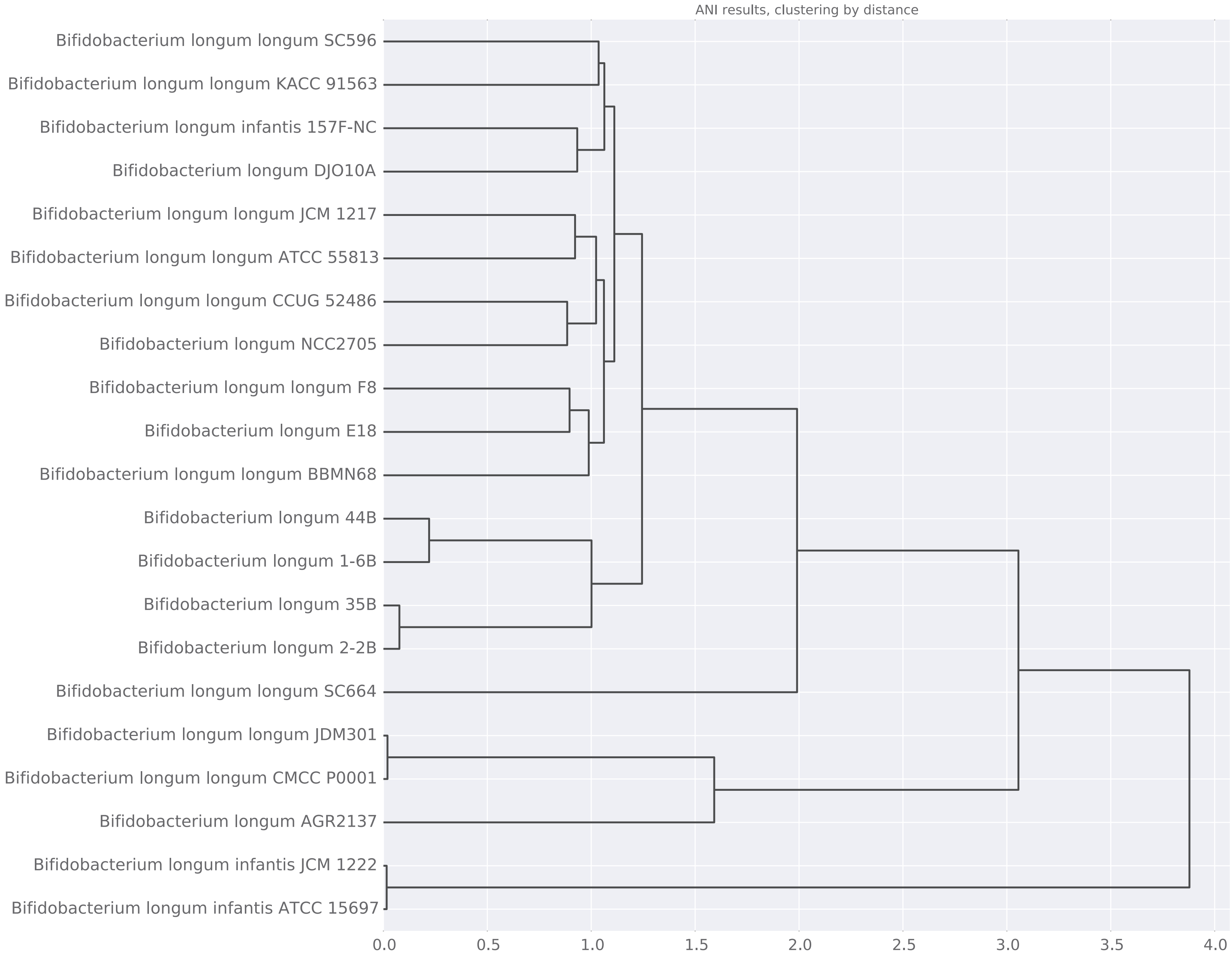
ANI results, clustering by distance
Bifidobacterium longum longum SC596
Bifidobacterium longum longum KACC 91563
Bifidobacterium longum infantis 157F-NC
Bifidobacterium longum DJO10A
Bifidobacterium longum longum JCM 1217
Bifidobacterium longum longum ATCC 55813
Bifidobacterium longum longum CCUG 52486
Bifidobacterium longum NCC2705
Bifidobacterium longum longum F8
Bifidobacterium longum E18
Bifidobacterium longum longum BBMN68
Bifidobacterium longum 44B
Bifidobacterium longum 1-6B
Bifidobacterium longum 35B
Bifidobacterium longum 2-2B
Bifidobacterium longum longum SC664
Bifidobacterium longum longum JDM301
Bifidobacterium longum longum CMCC P0001
Bifidobacterium longum AGR2137
Bifidobacterium longum infantis JCM 1222
Bifidobacterium longum infantis ATCC 15697
0.0
0.5
1.0
1.5
2.0
2.5
3.0
3.5
4.0

Figure S5

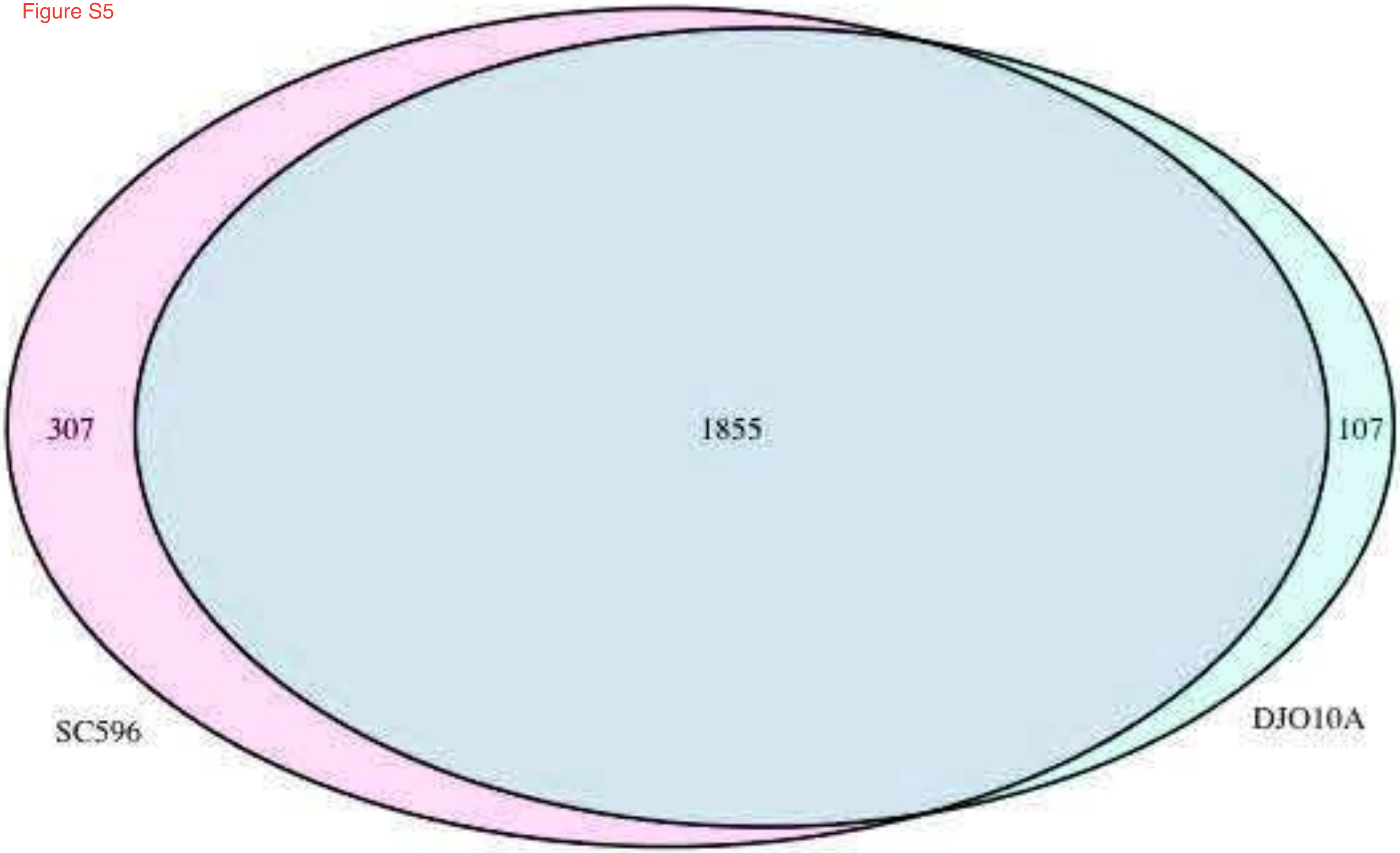

Figure S6

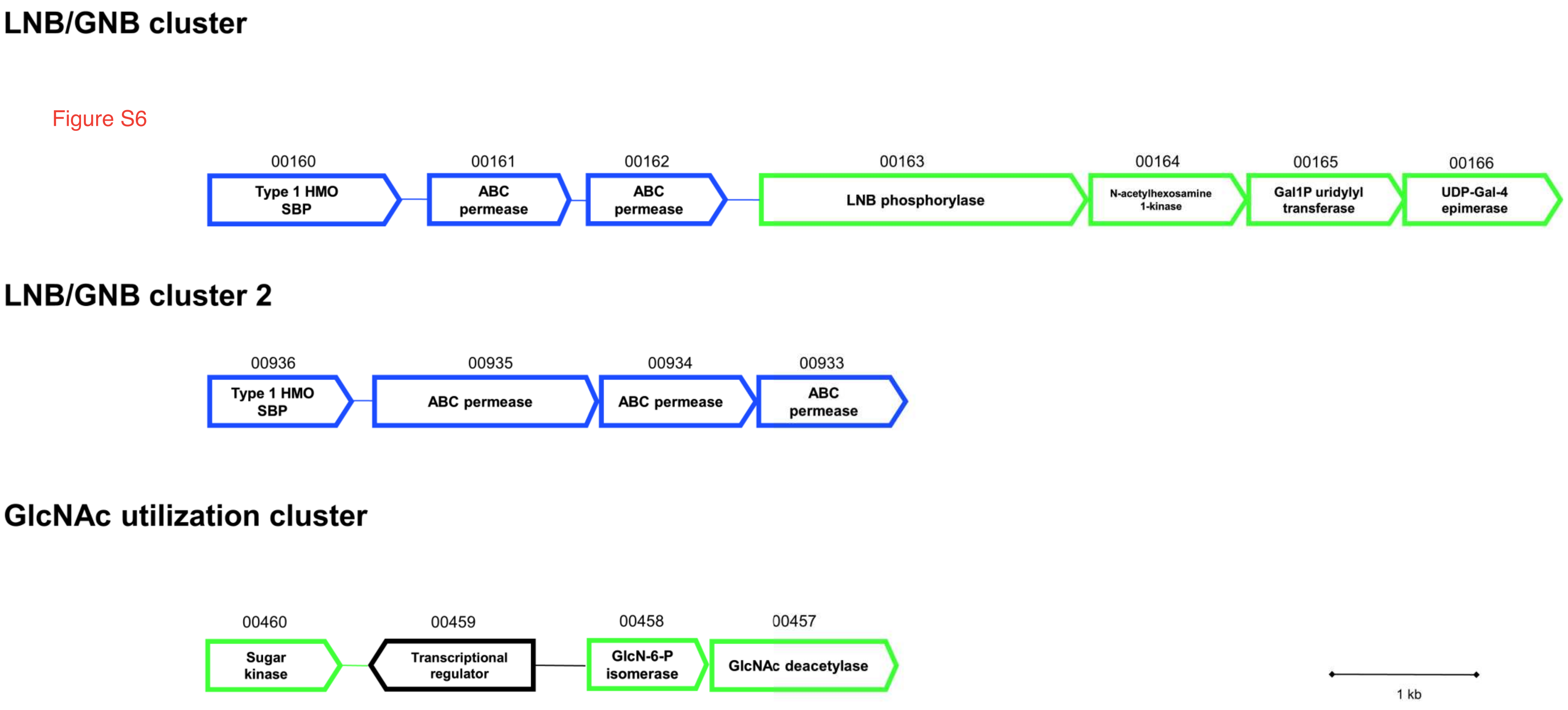
LNB/GNB cluster
00160
Type 1 HMO SBP
00161
ABC permease
00162
ABC permease
00163
LNB phosphorylase
00164
N-acetylhexosamine 1-kinase
00165
Gal1P uridylyl transferase
00166
UDP-Gal-4 epimerase
LNB/GNB cluster 2
00936
Type 1 HMO SBP
00935
ABC permease
00934
ABC permease
00933
ABC permease
GlcNAc utilization cluster
00460
Sugar kinase
00459
Transcriptional regulator
00458
GlcN-6-P isomerase
00457
GlcNAc deacetylase
1 kb
